# Neuropsychological performance in solvent-exposed vehicle collision repair workers in New Zealand

**DOI:** 10.1371/journal.pone.0189108

**Published:** 2017-12-13

**Authors:** Samuel Keer, Bill Glass, Dave McLean, Elizabeth Harding, Duncan Babbage, Janet Leathem, Yanis Brinkmann, Bradley Prezant, Neil Pearce, Jeroen Douwes

**Affiliations:** 1 Centre for Public Health Research, Massey University, Wellington, New Zealand; 2 Centre for eHealth & Centre for Person Centred Research, Auckland University of Technology, Auckland, New Zealand; 3 School of Psychology, Massey University, Wellington, New Zealand; 4 Department of Medical Statistics, London School of Hygiene and Tropical Medicine, London, United Kingdom; University of North Carolina at Chapel Hill, UNITED STATES

## Abstract

**Objectives:**

To assess whether contemporary solvent exposures in the vehicle collision repair industry are associated with objectively measured neuropsychological performance in collision repair workers.

**Methods:**

The RBANS battery and additional tests were administered to 47 vehicle collision repair and 51 comparison workers randomly selected from a previous questionnaire study.

**Results:**

Collision repair workers performed lower on tests of attention (digit span backwards: -1.5, 95% CI -2.4, -0.5; digit span total: -1.7, CI -3.3, -0.0; coding: -6.1, CI -9.9, -2.8; total attention scale: -9.3, CI -15.9, -2.8) and the RBANS total scale (-5.1, CI -9.1, -1.2). Additional tests also showed deficits in visual attention and reaction time (Trails B: -11.5, CI -22.4, -0.5) and motor speed/dexterity (coin rotation dominant hand & non-dominant: -2.9, CI -5.3, -0.4 and -3.1, CI -5.6, -0.7 respectively). The strongest associations were observed in panel beaters. Applying dichotomised RBANS outcomes based on the lowest percentile scores of a normative comparison group showed strongly increased risks for attention (5th percentile: OR 20.1, 95% CI 1.5, 263.3; 10th percentile: 8.8, CI 1.7, 46.2; and 20th percentile: 5.1, CI 1.5, 17.6, respectively). Those employed in the industry for ≤ 17 years (the median work duration) generally had lower scores in the attention domain scale and RBANS total scale compared to those employed >17 years suggesting a healthy worker survivor bias, but trends were inconsistent for other domains.

**Conclusions:**

This study has found significant deficits in cognitive performance in collision repair workers despite low current airborne exposures in New Zealand.

## Introduction

Millions of tons of organic solvents are produced globally each year and used in many industrial processes,[1] including the automotive repair industry where they are used extensively in raw form, and in bodywork fillers and various spray coatings. Workers exposed to solvents, through inhalation and dermal absorption, are at risk of developing symptoms of neurotoxicity including memory impairment, changes in personality and deficits in cognitive function.[2, 3] Prolonged exposure has also been associated with chronic solvent-induced encephalopathy (CSE, a condition caused by long-term solvent exposure and characterised by symptoms associated with central nervous system depression and deficits in neurobehavioural performance, which may persist even upon cessation of exposure[4]), as reported in automotive repair workers [5, 6] and other occupations. [7–9]

In a recent questionnaire survey of 370 collision repair workers and 211 comparison workers, we reported that collision repair workers in New Zealand continue to have a significantly increased risk of symptoms of neurotoxicity, including neurological (e.g. numbness, tingling or decreased sensation in extremities, dropping things unintentionally, balance problems, etc.), mood, memory, and concentration symptoms.[10] This is despite considerable changes in technology and health and safety practices in this industry and an associated decline in solvent exposures over the past 2 decades.[11] However, it is unclear whether these effects extend to cognitive deficits as measured by objective neuropsychological tests. Previous studies using these tests have reported reductions in attention span and sustained attention, immediate and delayed memory and motor speed.[5, 7, 8, 12] However, the findings have been inconsistent with some studies showing no association, suggesting that workers may be at risk of only ‘mild’ symptoms.[13–16] Alternatively, the inconsistent results may be due to small sample sizes for most studies, inadequate control for confounding, insufficiently sensitive neuropsychological tests,[17] or underestimation of the importance of relatively small average changes measured across workers.[18]

In the current study a neuropsychological test battery was administered to a randomly selected subset of collision repair (n = 47) and comparison workers (n = 51) who participated in our previous questionnaire study [10] in order to assess associations between contemporary mixed-solvent exposures and objectively measured neuropsychological performance.

## Methods

### Study population

The study participants comprised a random sample of 47 collision repair and 51 comparison workers from our previous questionnaire survey, the methods and results of which have been described elsewhere.[10] In brief, 370 collision repair workers (spray painters, panel beaters or auto-body repair workers, and office staff) were recruited from workshops throughout the north island of New Zealand. Office staff (n = 46) were all ex-tradesmen and were recoded as a spray painter or panel beater according to their previous job title, as this more accurately reflected their working life exposures; also the majority still performed some repair work, especially during busy periods, suggesting that, at least occasionally, they are at risk of being exposed.[10] A comparison group of 211 construction workers with negligible/no exposure to solvents was recruited in the same regions using a similar strategy. Informed written consent was obtained from all participants for involvement in each stage of the study and the study protocol was approved by the New Zealand Multiregional Ethics Committee (Application MEC/10/08/081). A sample of participants from the previous study (69 collision repair and 80 comparison workers) was re-contacted with the aim of recruiting at least 50 collision repair and 50 comparison workers for neuropsychological testing. The group size was based on power calculations conducted by Hooisma, Hanninen [19] which indicated that approximately 50 participants per group was sufficient to detect meaningful differences for individual tests comparable to those used in the current study (see below).

Seven collision repair workers and 20 comparison workers declined to participate, leaving a total of 59 (90%) collision repair and 60 (75%) comparison workers. Of those, ten collision repair and eight comparison workers met the exclusion criteria i.e., current recreational/sedative/anti-anxiety prescription drug use, history of major head trauma, and/or history of neurological or neurodegenerative disease including meningitis. Women (2 collision repair workers and 1 comparison worker) were also excluded due to low numbers. This resulted in complete test results being available for 47 collision repair workers (34 spray painters and 13 panel beaters) and 51 comparison workers.

### Neuropsychological test battery

Prior to testing, participants completed a brief questionnaire on issues likely to affect test performance (e.g., sleep, alcohol consumption and drug use in the past 48 hours). To ensure a uniform testing environment, all tests were conducted in a mobile station at the participant’s workplace by a single examiner. Tests were conducted throughout the day.

The test battery consisted of a modified (see below) version of the Repeatable Battery for the Assessment of Neuropsychological Status (RBANS, NCS Pearson Ltd, MN, USA).[20] The battery includes tests of immediate memory, visuospatial and construction skills, language, attention and delayed memory. The immediate memory tasks include a verbal serial list learning task (list of words) and recall of a short story. The shortened version of the Digit Span test for attention and working memory was substituted for the full version of the test[21] to improve test sensitivity and comparability with other studies. A coding task similar to the Digit Symbol Modalities test[22] was included to cover attention, visual scanning, tracking and motor speed. Tasks assessing language included both a picture naming and semantic fluency exercise. Assessment of delayed memory involved the recall of words or figures from earlier sections of the test after an intermission involving other, unrelated tasks. Also, a list recognition test was included to assess prompted delayed memory.

In addition to the RBANS, the following tests were administered: Trails A and B[23] and the Stroop Colour-Word test (Stoelting Co., Illinois, USA),[24] to measure aspects of cognitive function associated with attention, reaction time and processing speed; the Coin Rotation Test for motor speed and dexterity;[25] the Rey 15 item test for assessment of malingering/symptom validity;[26] the National Adult Reading Test (NART) for verbal intelligence;[27] and the Depression, Anxiety and Stress Scale (DASS).[28] The last three tests were included to assess participant effort and to allow for test analyses to be adjusted for premorbid intelligence and/or psychological factors that may influence test performance[12] Completed tests were scored twice, once by the test examiner and once by a clinical psychologist who was blinded to the occupation of the test subjects. Any inter-rater discrepancies were discussed and resolved before test results were entered into the database.

Information on demographics and other potential confounders used in the analyses was collected by questionnaire in our previous study. [10]

### Exposure groups

Workers were stratified according to their current job title (i.e. spray painter, panel beater), except office workers (as described above). Also, one panel beater in the current study reported working for a defined period as a spray painter in the past and was recoded accordingly. Job title was subsequently used as a proxy of current and previous exposure combined i.e. for the majority of workers job title had not changed since they had commenced working in this industry.

We assessed current airborne solvent exposure by conducting full-shift airborne personal exposure measurements in a random sample of spray painters (n = 50) and panel beaters (n = 36) and a small group of office workers (n = 6) with no history of exposure work (these workers were not included in the previous questionnaire survey or current study). Results (and detailed methods) are described elsewhere (10) and briefly discussed in the discussion of the current paper.

### Statistical analyses

Test results were compared between collision repair and comparison workers for individual test scores, combined ‘scale’ scores for each neurobehavioural domain and for the RBANS ‘total scale’ score (reflecting overall performance on the RBANS) using linear regression. The ‘scaled’ scores are the combined score for each domain normalised based on the participant’s age, as per RBANS guidelines [20]. For Trails A and B, coin rotation, 15 Item, NART, and DASS tests raw scores were used (with appropriate adjustment for age in the analyses, see below). For the Stroop test the ‘interference’ score was used, consistent with international guidelines[29]. In addition to analysing RBANS scores on a continuous scale we also used dichotomised scores based on cut-points representing the lowest 5^th^, 10^th^ and 20^th^ percentile scores of an RBANS normative comparison group[30] for each of the domain ‘scaled’ scores. These approximate RBANS definitions of ‘Low’, ‘Borderline’ and ‘Low average’ test performance.[30] For analyses comparing dichotomised outcomes we used logistic regression.

Regression analyses for individual test results were adjusted for age, ethnicity, smoking status, alcohol consumption in the past 48 hours, depression, anxiety and stress (DASS), premorbid verbal intelligence (NART), malingering/participant effort (15 Item), test time during the day, and day of week.[31] We did not adjust for age for the scaled domain and total scores as these are already normalised for age. Other potential confounders including different measures of alcohol consumption (lifetime frequency and lifetime drinks per week), sleep quality, chronic diseases (e.g., diabetes), minor head injuries/concussion, chronic fatigue and pre-existing health issues were also considered, but these did not appreciably affect the observed associations (Data not reported). As a higher score on the Trail Making Tests (A and B) indicates lower performance, the algebraic signs of the coefficients and confidence intervals for these variables were inverted for ease of interpretation. For the analyses by percentile groups we adjusted for a more restricted set of potential confounders (ethnicity, test time during the day and day of week, alcohol consumption in the past 48 hours, smoking status, depression, anxiety and stress (DASS) and verbal intelligence (NART)) as small numbers in some strata did not permit for adjustment for all potential confounders.

The effect of employment duration was assessed by dichotomising collision repair workers based on median work duration i.e., those who had worked in the industry for ≤17 years (average of 10.5 years; range 5.4–16.2; n = 23) and those who had worked in the industry for >17 years (average 28.3 years; range 16.8–50; n = 24). Due to the high correlation of age with employment duration (Spearman’s correlation coefficient = 0.92) and the resulting potential for multicollinearity, a second regression for the individual test scores (domain scores are already scaled for age) was conducted controlling for all confounders except age, and results were compared with those of the full regression model including age. Due to the low number of panel beaters (n = 13) stratified analyses for spray painters and panel beaters were not conducted for comparisons between the two employment duration groups; instead, the analyses were adjusted for job title (spray painter/panel beater).

## Results

Māori and Pacific people were underrepresented in the collision repair workers compared to the comparison group (Māori, 6% vs 25%, p<0.05; Pacific, 4% vs.16%, p<0.05) (Table 1). Collision repair workers were also less likely to smoke (28% vs 33%, p = 0.55) or to have had a tertiary education (4% vs. 12%, p = 0.17) and generally scored lower on the depression, anxiety and stress scale (Depression, 4.4 vs. 5.1, p = 0.14, anxiety, 3.4 vs. 5.1, p<0.1, stress, 8.6 vs. 10.6, p = 0.13), but this did not reach statistical significance. Panel beaters were on average older (not statistically significant) than both spray painters (45.1 vs 38.8 years, p<0.1) and the comparison group (45 vs 39.0 years, p = 0.13). Collision repair workers scored lower on the National Adult Reading Test (not statistically significant) for premorbid intelligence (19.2 vs 18.1 errors, p = 0.62). All analyses were controlled for these potential confounders.

**Table 1:** Characteristics of study population.

	Comparison group (n = 51)		All Collision repair (n = 47)		Panel beaters (n = 13)		Spray painters (n = 34)	
	n	%	n	%	n	%	n	%
**Ethnicity**								
Māori	13	25	3	6	0	0	3	8
Pacific	8	16	2	4	1	8	1	3
European New Zealanders and others	30	59	42**	89	12	92	30	88
**Smoking Status**								
Non-smoker	18	35	19	40	3	23	16	47
Ex-smoker	16	31	15	32	6	46	9	26
Current smoker	17	33	13	28	4	31	9	26
**Lifetime alcohol (frequency)**								
Never	1	2	1	2	0	0	1	3
Less than once month	8	16	4	9	2	15	2	6
1–2 times week	25	49	25	53	6	46	19	56
3–5 times week	14	27	16	34	5	38	11	32
Daily	3	6	1	2	0	0	1	3
**Education level**								
primary school	0	0	2	4	0	0	2	6
secondary school	36	71	38	81	11	85	27	79
trade certification	9	18	5	11	2	15	3	9
Tertiary/University	6	12	2	4	0	0	2	6
	**Mean**	**Range**	**Mean**	**Range**	**Mean**	**Range**	**Mean**	**Range**
**Age**	39.0	19–65	37.8	21–62	45.1	32–64	38.8	24–60
**Lifetime Alcohol (Mean drinks per week)**	15.8	0–100	14.3	0–50	12.4	1–36	15.1	0–50
**Alcohol in past 48 hours (mean drinks)**	2.5	0–42	3.3	0–40	2.5	0–8	3.6	0–40
**Duration of employment (Yrs)**	-	-	19.6	5.4–50.0	22.5	12.6–46.6	18.5	5.4–50.0
**Validation Test Scores**	**Mean**	**SD**	**Mean**	**SD**	**Mean**	**SD**	**Mean**	**SD**
15 item	14.1	1.7	13.2	2.2	13.6	1.6	13.1	2.4
NART	18.1	10.8	19.2	10.4	22.8	11.3	17.8	9.8
DASS Depression	5.1	4.6	3.8	4.4	2.9	2.8	4.1	4.9
DASS Anxiety	5.1	4.7	3.6^	3.4	2.8	3.1	3.9	3.4
DASS Stress	10.6	6.7	8.6	6.1	8.8	5.2	8.5	6.5

^ = p<0.1,

** = p<0.01 (Students t-test)

Collision repair workers performed significantly lower on tests reflecting attentional performance (Table 2). In particular, significant deficits were observed for the digit span backwards (-1.5; 95% CI -2.4, -0.5), digit span total (-1.7; CI -3.3, -0.0), coding (-6.1; CI 9.9, -2.2) and the aggregate total attention scale (-9.3; CI -15.9, -2.8). The overall RBANS total scale was also lower for collision repair workers (-5.1; CI -9.1, -1.2) with the most pronounced deficit observed for panel beaters. Collision repair workers also performed lower for the additional tests including dominant and non-dominant hand coin rotation (-2.9; CI -5.3, -0.4 and -3.1; CI -5.6, -0.7, respectively) and Trail Making Test B (-11.5; CI -22.4, -0.5) with the strongest effects observed in panel beaters (Table 2).

**Table 2:** Neuropsychological test scores for comparison and collision repair workers.

	Comparison group	All Collision repair		Panel Beaters		Spray painters	
RBANS battery	(n = 51)	(n = 47)		(n = 13)		(n = 34)	
	Mean (SD)	Mean (SD)	Difference (95%CI)	Mean (SD)	Difference (95%CI)	Mean (SD)	Difference (95%CI)
***Immediate memory***							
RBANS 1 (list learning)	29.6 (4.0)	28.4 (4.5)	-0.8 (-2.4, 0.8)	27.4 (4.6)	**-2.0 (-4.2, 0.2)**^	28.8 (4.4)	-0.2 (-2.0, 1.5)
RBANS 2 (story memory)	16.9 (3.6)	15.7 (3.9)	-1.3 (-2.8, 0.3)	16.7 (3.1)	-0.9 (-3.1, 1.3)	15.3 (4.2)	**-1.4 (-3.2, 0.3)**^
Total scale Immediate Memory	95.6 (12.6)	91.9 (13.9)	-2.5 (-7.8, 2.9)	93.5 (9.3)	-5.0 (-12.7, 2.7)	91.3 (15.3)	-1.3 (-7.2, 4.5)
***Visuospatial/Construction***							
RBANS 3 (figure copy)	17.1 (2.5)	17.8 (1.8)	0.6 (-0.4, 1.5)	17.5 (1.7)	0.3 (-1.0, 1.7)	17.9 (1.9)	0.7 (-0.3, 1.8)
RBANS 4 (line orientation)	18.8 (1.9)	18.7 (2.1)	-0.1 (-1.0, 0.8)	19.3 (1.44)	0.5 (-0.8, 1.8)	18.4 (2.3)	-0.4 (-1.4, 0.6)
Total scale vis./const.	99.6 (15.8)	99.7 (15.2)	-2.2 (-8.9, 4.5)	100.2 (16.0)	-3.6 (-13.2, 6.0)	99.5 (15.1)	-1.6 (-8.9, 5.7)
***Language***							
RBANS 5 (picture naming)	9.5 (2.0)	10.0 (0.0)	0.2 (-0.5, 0.8)	10.0 (0.0)	0.3 (-0.6, 1.1)	10.0 (0.0)	0.1 (-0.6, 0.8)
RBANS 6 (semantic fluency)	21.5 (5.2)	21 (3.9)	-1.0 (-3.0, 1.0)	20.5 (3.7)	-1.7 (-4.6, 1.2)	21.2 (4.0)	-0.7 (-2.9, 1.5)
Total scale Language	98.1 (15.0)	97.2 (12.0)	-2.4 (-8.3, 3.4)	97.1 (8.1)	-3.6 (-12.0, 4.8)	97.2 (13.3)	-2.0 (-8.4, 4.5)
***Attention***							
RBANS 7a (digit span forward)	10.5 (2.3)	10.3 (2.4)	-0.2 (-1.2, 0.8)	9.9 (2.3)	-1.1 (-2.6, 0.3)	10.5 (2.5)	0.2 (-0.9, 1.3)
RBANS 7b (digit span backward)	7.8 (2.3)	6.1 (2.0)	-1.5 (-2.4, -0.5)**	6.1 (1.6)	-1.8 (-3.2, -0.4)*	6.1 (2.2)	-1.3 (-2.4, -0.2)*
RBANS 7c (digit span total)	18.2 (4.1)	16.5 (3.7)	-1.7 (-3.3, -0.0)*	16.0 (2.7)	-2.9 (-5.3, -0.6)*	16.7 (4.1)	-1.1 (-2.9, 0.8)
RBANS 8 (coding)	50.6 (9.4)	46.1 (8.4)	-6.1 (-9.9, -2.2)**	45.0 (6.9)	-7.7 (-13.2, -2.2)*	46.5 (8.9)	-5.3 (-9.6, -1.0)*
Total scale Attention	94.6 (14.2)	88.6 (16.2)	-9.3 (-15.9, -2.8)**	87.7 (11.4)	-13.1 (-22.5, -3.7)**	88.9 (17.8)	-7.7 (-14.8, -0.5)*
***Delayed Memory***							
RBANS 9 (list recall)	7.0 (1.7)	5.7 (2.2)	-1.0 (-1.7, -0.3)**	5.4 (2.3)	**-1.0 (-2.0, 0.1)**^	5.8 (2.2)	-1.0 (-1.8, -0.2)*
RBANS 10 (list recognition)	19.6 (1.7)	19.6 (0.6)	0.0 (-0.6, 0.6)	19.6 (0.7)	0.0 (-0.9, 0.9)	19.6 (0.6)	0.0 (-0.7, 0.7)
RBANS 11 (story recall)	9.2 (2.2)	8.4 (2.5)	-0.6 (-1.5, 0.3)	8.8 (2.4)	-0.3 (-1.6, 1.0)	8.2 (2.5)	-0.8 (-1.8, 0.2)
RBANS 12 (figure recall)	14.2 (3.4)	13.9 (3.1)	0.0 (-1.4, 1.5)	13.4 (3.3)	-0.3 (-2.4, 1.8)	14.0 (3.1)	0.2 (-1.4, 1.8)
Total scale Delayed Memory	96.8 (8.4)	93.3 (8.5)	-1.4 (-5.4, 2.5)	94.3 (8.1)	-1.6 (-7.3, 4.1)	92.9 (8.7)	-1.4 (-5.7, 3.0)
***RBANS total scale***	96.4 (10.1)	92.0 (10.5)	-5.1 (-9.1, -1.2)*	91.9 (7.1)	-7.8 (-13.4, -2.2)**	92.0 (11.7)	**-4.0 (-8.3, -0.3)**^
**Additional Tests**							
***Visual Attention/Reaction Time***							
Trails Aˠ	23.8 (9.9)	24.4 (6.9)	-2.0 (-5.6–1.6)	28.15 (8.1)	-5.6 (- 10.6, -0.6)*	22.9 (5.8)	-0.3 (- 4.2, 3.6)
Trails Bˠ	68.1 (29.1)	73.4 (27.7)	-11.5 (- 22.4, -0.5)*	67.4 (22.1)	-6.4 (- 22.0–9.2)	75.8 (29.5)	-13.9 (-26.1, -1.8)*
Stroop (I)	2.0 (10.7)	0.5 (7.3)	-3.1 (-7.4–1.2)	-1.5 (6.7)	-4.4 (-10.6–1.7)	1.2 (7.5)	-2.5 (-7.3, 2.3)
***Motor speed/Dexterity***							
Coin rot. Dominant hand	33.7 (5.3)	31.9 (6.2)	-2.9 (-5.3, -0.4)*	30.00 (6.3)	-4.0 (-7.5, -0.5)*	32.6 (6.1)	**-2.3 (-5.0, -0.4)**^
Coin rot. Non-dominant	31.3 (5.2)	28.2 (5.7)	-3.1 (-5.6, -0.7)*	26.69 (5.0)	-4.3 (-7.8, -0.8)*	28.8 (5.8)	**-2.6 (-5.3, -0.2)**^

^ = p<0.1,

* = p<0.05,

** = p<0.01

Adjusted for age, ethnicity, alcohol consumption in the past 48 hours, smoking status, DASS A, S and D, test time (of day) and test day (of week), symptom validity/malingering (Rey 15 item) and premorbid intelligence (NART).

^ˠ^Trails A and B—time to complete each test, therefore higher score represents poorer performance on test—Algebraic sign of coefficient changed accordingly

Analyses based on the RBANS definitions of ‘low’ (5^th^ percentile), ‘borderline’ (10^th^ percentile) and ‘low average’ (20^th^ percentile) test performance showed a similar pattern, and highlight that observed deficits are relatively large especially for the domain of attention (Table 3). In particular, for collision repair workers we found a twenty-fold (p<0.05) increased risk for “low” test results after adjusting for potential confounders, although due to small numbers in some strata confidence limits were wide. Also, although crude unadjusted analyses (Table 3) did show increased risks, the magnitude of the effects was considerably lower. This difference was largely due to adjustment for ethnicity; subsequent sensitivity analyses excluding Māori and Pacific people resulted in similarly high odds ratios (S1 Table) suggesting that adjusted risk estimates are robust.

**Table 3:** Neuropsychological test scores based on the bottom 5^th^, 10^th^ and 20^th^ percentiles for comparison and collision repair workers.

	Comparison group	All Collision repair		
	(n = 51)	(n = 47)		
			Unadjusted OR	Adjusted OR
RBANS battery	N (%)	N (%)	(95%CI)	(95%CI)
***Immediate memory***				
5th percentile	2 (3.9)	4 (8.5)	2.3 (0.4, 13.1)	-
10th percentile	4 (7.8)	10 (21.3)	**3.2 (0.9, 10.9)**^	11.6 (1.4, 99.7)*
20th percentile	17 (33.3)	21 (44.7)	1.6 (0.7, 3.7)	2.0 (0.6, 6.8)
***Visuospatial/Construction***				
5th percentile	4 (7.8)	1 (2.1)	0.3 (0.0, 2.4)	-
10th percentile	5 (9.8)	4 (8.5)	0.9 (0.2, 3.4)	6.3 (0.2, 166.2)
20th percentile	13 (25.5)	13 (27.7)	1.1 (0.5, 2.7)	1.9 (0.6, 6.3)
***Language***				
5th percentile	3 (5.9)	1 (2.1)	0.3 (0.0, 3.5)	-
10th percentile	5 (9.8)	3 (6.4)	0.6 (0.1, 2.8)	5.7 (0.1, 259.7)
20th percentile	7 (13.7)	9 (19.2)	1.5 (0.5, 4.4)	**4.5 (0.9, 22.3)**^
***Attention***				
5th percentile	4 (7.8)	11 (23.4)	3.6 (1.1, 12.2)*	20.1 (1.5, 263.3)*
10th percentile	8 (15.7)	15 (31.9)	**2.5 (1.0, 6.7)**^	8.8 (1.7, 46.2)*
20th percentile	12 (23.5)	22 (46.8)	2.9 (1.2, 6.8)*	5.1 (1.5, 17.6)**
***Delayed Memory***				
5th percentile	1 (2.0)	2 (4.3)	2.2 (0.2, 25.3)	-
10th percentile	2 (3.9)	2 (4.3)	1.1 (0.1, 8.1)	-
20th percentile	4 (7.8)	11 (23.4)	3.6 (1.1, 12.2)*	3.0 (0.7, 12.2)
***RBANS total scale***				
5th percentile	2 (3.9)	1 (2.1)	0.5 (0.0, 6.1)	-
10th percentile	5 (9.8)	5 (10.6)	1.1 (0.3, 4.1)	6.3(0.4, 90.7)
20th percentile	9 (17.7)	17 (36.2)	2.6 (1.0, 6.7)*	14.1 (2.4, 83.6)**

^ = p<0.1,

* = p<0.05,

** = p<0.01

Adjusted for ethnicity, alcohol in the past 48 hours, smoking status, DASS A, S and D, NART, test time (of day) and test day (of week).

Those with shorter employment duration (i.e. <17 years) performed lower on the domain scale score for attention (-20.6; 95% CI -30.6, -10.6, versus -6.8; CI -16.5, 2.8) as well as the non-dominant hand coin rotation task (-6.5; CI -10.4, -2.6 versus -2.3; CI -6.2, 1.5, Table 4). However, the trend of lower performance in those with shorter employment duration was not observed consistently across different domains with tests of immediate and delayed memory showing somewhat more pronounced effects in workers with longer employment duration. Adjusting for age altered the effect measures for some outcomes, but the effect on trends was negligible when compared to the model excluding age (S2 Table).

**Table 4:** Neuropsychological test scores for collision repair workers stratified by employment duration.

		Employment Duration (mean years)			
	Comparison group	< 17 years (10.5)		>17 years (28.4)	
	(n = 51)	(N = 23)		(n = 24)	
	Mean (SD)	Mean (SD)	Difference (95%CI)	Mean (SD)	Difference (95%CI)
***Immediate memory***					
RBANS 1 (list learning)	29.6 (4.0)	30.8 (4.2)	-0.9 (-3.4, 1.7)	26.2 (3.5)	-3.0 (-5.5, -0.6)*
RBANS 2 (story memory)	16.9 (3.6)	15.7 (4.4)	-1.1 (-3.6, 1.5)	15.6 (3.5)	-0.8 (-3.3, 1.7)
Total scale Immediate Memory	95.6 (12.6)	93.2 (16.8)	-3.4 (-12.1, 5.2)	90.7 (10.6)	-6.3 (-14.7, 2.0)
***Visuospatial/Construction***					
RBANS 3 (figure copy)	17.1 (2.5)	18.0 (1.5)	0.1 (-1.5, 1.6)	17.6 (2.1)	0.5 (-1.0, 2.1)
RBANS 4 (line orientation)	18.8 (1.9)	18.9 (2.2)	0.7 (-0.7, 2.2)	18.5 (2.1)	0.2 (-1.2, 1.7)
Total scale vis./const.	99.6 (15.8)	97.4 (14.2)	-5.0 (-15.8, 5.9)	101.9 (16.1)	-2.5 (-13.0, 8.1)
***Language***					
RBANS 5 (picture naming)	9.5 (2.0)	10 (0.0)	0.2 (-0.8, 1.2)	10.0 (0.0)	0.3 (-0.7, 1.3)
RBANS 6 (semantic fluency)	21.5 (5.2)	21.6 (4.4)	-1.8 (-5.1, 1.5)	20.4 (3.4)	-1.6 (-4.8, 1.6)
Total scale Language	98.1 (15.0)	99.1 (11.3)	-1.7 (-11.2, 7.8)	93.4 (12.6)	-5.1 (-14.3, 4.0)
***Attention***					
RBANS 7a (digit span forward)	10.5 (2.3)	10.2 (2.3)	**-1.6 (-3.2, 0.1)**^	10.5 (2.5)	-0.8 (-2.3, 0.8)
RBANS 7b (digit span backward)	7.8 (2.3)	6.4 (2.0)	-1.8 (-3.4, -0.2)*	5.8 (2.0)	-1.9 (-3.5, -0.3)*
RBANS 7c (digit span total)	18.2 (4.1)	16.7 (3.6)	-3.3 (-6.0, -0.5)*	16.3 (3.9)	**-2.6 (-5.3, 0.1)**^
RBANS 8 (coding)	50.6 (9.4)	46.4 (8.0)	-10.0 (-16.3, -3.8)**	45.8 (8.9)	**-5.6 (-11.7, 0.5)**^
Total scale Attention	94.6 (14.2)	82.3 (14.2)	-20.6 (-30.6, -10.6)**	94.6 (16.0)	-6.8 (-16.5, 2.8)
***Delayed Memory***					
RBANS 9 (list recall)	7.0 (1.7)	7.2 (1.3)	0.0 (-1.1, 1.2)	4.3 (1.9)	-1.9 (-3.0, -0.8)
RBANS 10 (list recognition)	19.6 (1.7)	19.8 (0.4)	0.1 (-0.9, 1.1)	19.5 (0.7)	-0.1 (-1.1, 0.8)
RBANS 11 (story recall)	9.2 (2.2)	9.3 (2.2)	0.3 (-1.1, 1.8)	7.5 (2.5)	-0.8 (-2.3, 0.6)
RBANS 12 (figure recall)	14.2 (3.4)	15.4 (2.5)	0.6 (-1.7, 3.0)	12.3 (3.0)	-1.1 (-3.4, 1.1)
Total scale Delayed Memory	96.8 (8.4)	95.5 (6.3)	0.9 (-5.4, 7.3)	91.1 (9.8)	-3.7 (-9.9, 2.4)
RBANS total scale^	96.4 (10.1)	91.0 (11.5)	-8.7 (-15.1, -2.4)**	92.9 (9.7)	-7.0 (-13.2, -0.9)*
**Additional Tests**					
***Visual Attention/Reaction Time***					
Trails Aˠ	23.8 (9.9)	22.8 (6.6)	-7.0 (- 12.7, 1.2)	25.8 (6.9)	-4.4 (- 10.0, 1.2)
Trails Bˠ	68.1 (29.1)	71.2 (27.9)	-11.0 (- 28.9, 6.9)	75.6 (27.9)	-2.3 (- 19.8, 15.2)
Stroop (I)	2.0 (10.7)	3.0 (7.4)	-2.4 (-9.4, 4.7)	-1.9 (6.6)	**-6.3 (-13.2, 0.6)**^
***Motor speed/Dexterity***					
coin rot. Dominant hand	33.7 (5.3)	32.0 (6.7)	-	31.9 (5.8)	**3.3 (-0.4, 7.0)**^
coin rot. Non-dominant hand	31.3 (5.2)	27.6 (6.7)	-6.5 (-10.4, -2.6)**	28.8 (4.5)	-2.3 (-6.2, 1.5)

^ = p<0.1,

* = p<0.05,

** = p<0.01

Adjusted for age, ethnicity, alcohol consumption in the past 48 hours, Job title (spray painter/panel beater), smoking status, DASS A, S and D, test time (of day) and test day (of week), malingering/symptom validity (Rey 15 item) and premorbid intelligence (NART).

^ˠ^Trails A and B—time to complete each test, therefore higher score represents poorer performance on test—Algebraic sign of coefficient changed accordingly

## Discussion

Collision repair workers performed significantly lower in the domains of attention, visual attention/reaction time, motor speed/dexterity and, to a lesser extent, memory, with lowest performance reported in panel beaters. No consistent difference with employment duration was found.

The findings of poorer test performance, particularly for attentional performance and memory are consistent with those of our previous questionnaire study [10], where workers reported significantly more symptoms indicative of memory/attention deficits (e.g. general forgetfulness, difficulty remembering names/dates/schedules, difficulty concentrating, absent-mindedness, confusion when concentrating etc.). The findings of lower attentional, memory and motor performance are also consistent with previous international studies in car painters and other solvent exposed workers.[5, 7, 8, 12, 32] including a large 2008 meta-analysis[12], which assessed the impact of solvent mixtures on neurobehavioural performance using the findings of 46 epidemiological studies conducted between 1976 and 2004, involving 53 groups of solvent-exposed workers (i.e. house, car or other industrial painters [5, 6, 19, 33]). Findings consistent with adverse effects of solvent exposures on neurobehavioural function were found for 43 of the 48 tests used; of those, 12 were statistically significant (p≤0.05). The strongest effects were shown for attention, with 40% of these tests demonstrating significant effects. Tests of memory, construction and motor speed also showed significant negative effects. Although the test results are not directly comparable, the domains most strongly affected in the meta-analysis were consistent with those affected in the current study. This was particularly the case for the attentional performance tests of Digit span and Trail making A and B. Furthermore, the meta-analysis showed that longer duration of solvent exposure was associated with reduced effect sizes in 7 of 12 tests, suggesting a healthy worker effect, similar to what we observed in the current study and our previous study. [10]

The deficit in motor speed and dexterity (Coin Rotation Task) observed in the current study may be suggestive of peripheral nervous system effects[34] which may also explain the significantly increased risk of ‘neurological’ symptoms (i.e., weakness, numbness or tingling in the extremities, dropping things unintentionally, sensorial changes, balance problems, etc.) observed in our previous questionnaire study.[10] Collision repair workers also performed lower on the RBANS total scale score, which is considered one of the measures least susceptible to natural variations in baseline performance,[35] suggesting that the differences in performance observed are likely due to differences in exposure rather than baseline performance.

The effects on neuropsychological performance appear to occur at airborne solvent levels below international exposure standards i.e., our previous survey, in which the current study was nested, found geometric mean concentrations for all solvents combined over a full-work shift of only 2.3 ppm in spray painters and 0.6 ppm in panel beaters. [10] Alternatively, effects observed in this study may be attributable to high historical exposures resulting in persistent deficits in performance.[36] However, this is unlikely to fully explain the lower performance observed in workers with shorter durations of employment (5–17 years), especially as significant reductions in solvent exposure levels in this industry are likely to have occurred as early as two decades ago.[11] It is also highly plausible, as we have suggested previously,[10] that airborne exposures do not accurately reflect the total body burden of solvent exposure and that dermal exposures (which we did not measure) may be more important. In fact, other studies have suggested that dermal solvent exposures may contribute >50% of the total body burden, particularly when respiratory protection is adequate.[37, 38] It is also possible that other exposures including heavy metals (e.g. lead), alcohol consumption and frequent use of vibrating tools may have contributed to at least some of the observed increased risks.[39–41] Although lead was used historically, it was largely phased out with the introduction of polyester resin-based fillers in the 1970’s. It is currently used occasionally in New Zealand for classic vehicle restorations, however, none of the shops involved in the current or previous study [10]) reported using lead. Alcohol consumption in the past 48 hours was adjusted for in the analyses as acute intoxication is associated with deficits in cognitive and motor performance.[42] However, recent consumption may not take into account the effects of long-term alcohol use.[43] Nonetheless, adjusting for lifetime rather than recent alcohol use had little effect on the results (Tables A-C in S3 Table and Tables A-C in S4 Table) and neither did adjusting for both simultaneously (S5 Table). Finally use of vibrating tools would account only for the lower performance in motor speed/manual dexterity and not for other test results for which scores were also lower.

Of the collision repair subgroups, panel beaters generally performed the lowest, which is similar to the findings of our previous questionnaire study. A study by Daniell, Stebbins (6] also reported an increased risk of cognitive deficits in panel beaters, but only in those who had previously worked as a spray painter. In our study, only seven panel beaters from the larger survey[10]—and one who completed the neuropsychological tests—reported having worked as a spray painter. These were recoded as spray painters for all analyses. The effects observed are therefore unlikely to be due to the misclassification of panel beaters and the reasons for more pronounced effects in this group therefore remain unclear, particularly since airborne exposures were lower than those in spray painters[10]. However, exposure assessment in our previous study did not include chlorinated solvents (perchloroethylene, tetrachloroethylene and methylene chloride) which have been associated with symptoms of neurotoxicity[44] and are a likely exposure source for panel beaters through the regular use of heavy duty cleaning and degreasing aerosol sprays. Alternatively, as noted above, dermal exposures may be of particular importance for panel beaters and could explain the stronger associations in this group.

Several studies have reported increased risks of subjective symptoms in collision repair and other solvent exposed workers, but did not find associations with objectively measured cognitive performance. This has led to the suggestion that contemporary low-level exposures may be associated with “only” low-grade nervous system dysfunction.[13–16] However, the results of our analyses focussing on more severe outcomes (i.e. dichotomised by lowest percentile cut-points based on the RBANS normative comparison group) suggest that such a conclusion may not be warranted. In particular, although the numbers were small in some strata, with correspondingly wide confidence intervals, collision repair workers were significantly more likely to score in the ‘low’, ‘borderline’ and ‘low average’ range for attention, in the ‘borderline’ range for immediate memory and in the ‘low average’ range for the RBANS total scale (Table 3). This is consistent with the findings of our previous questionnaire study which showed the most pronounced effects when using symptom cut-points reflecting a greater number of symptoms.[10]

For some tests and scaled scores the lowest performance was reported in those workers who had the shortest work duration (Table 4). This lack of a clear work duration-response trend is consistent with the findings of our previous study, where those with the longest employment duration (20+ years) reported fewer symptoms than those with medium duration (10–19 years).[10] Other studies also failed to find a clear work duration-response trend[9, 45] as demonstrated in a large meta-analysis.[12] This is likely due to healthy worker survivor bias[12] which results from workers who develop symptoms leaving the industry or moving to roles with lower exposures, with those remaining potentially being less susceptible to the effects of solvents or having a higher cognitive ‘reserve’.[46, 47] Further evidence for this was found when we repeated the analyses using outcomes defined as lowest percentile scores which showed particularly strong associations (albeit with wide confidence intervals) for attention in those who had been in the industry for ≤17 years (5^th^ percentile: OR = 53.3, 95% CI 3.3, 862.5; 10^th^ percentile: 12.5, CI 2.3, 68.6 and 20^th^ percentile: 21.1, CI 4.2, 105.9) compared to those who worked in this industry for >17 years (4.6, CI 0.3, 73.9; 4.3, CI 0.6, 28.8; and 1.2, CI 0.3, 5.5 respectively, (S5 Table).

Although workers were invited at random to take part in neuropsychological testing, thus representing a random sample of the workers involved in our previous study (n = 370) [10], this may not necessarily be representative of all collision repair workers in New Zealand. However, as noted in our previous study, workers were recruited to be representative of all workers in this industry, although this was not formally tested. Of those who were invited to take part in the neuropsychological testing, 90% of the collision repair workers and 75% of the comparison group agreed to participate. This is relatively high for these types of studies suggesting that selection bias, if present, is likely to be small. Analyses of demographic characteristics and subjective symptoms between responders and non-responders for both the current study and our previous study showed no differences between groups, confirming that selection bias is unlikely to be an issue. Some differences in alcohol consumption, ethnicity, duration of employment in the industry and the number of workers who had completed a trade certification were observed between those who were re-recruited for testing and those who were not; however differences were for the most part minor and the participant groups were otherwise representative of the previous study groups (S11 Table).

It is possible that some of the effects observed may be due to cross shift and/or cross-workweek exposures and are at least partially reversible. However, analyses controlling for when neuropsychological tests were conducted (time during the day and week) did not show appreciable differences in neuropsychological performance (Tables 2–4). Subsequent analyses stratified by time of the week (i.e. Monday-Wednesday versus Thursday/Friday) showed that performance was similarly low in those tested at the start of the week compared to those tested at the end of the week (S6 Table) suggesting that the influence of cross-week exposures (as opposed to long-term chronic exposure) on performance is small.

Some participants in the comparison group may have had occupational exposure to solvents and this is a potential limitation of the study. However, excluding workers who reported working regularly with solvents (n = 7) made little difference to the results (S7 Table). Also, we included office workers (n = 4) for whom we reclassified their job titles (see above) which may have resulted in misclassification. However, at the time of the study all four workers performed some repair work on the shop floor, especially during busy periods and therefore were likely to be at risk of at least some ‘current’ exposure. Analyses excluding them did not affect the results (S8 Table).

Differences in age, ethnicity, smoking, alcohol consumption, education, verbal intelligence and depression, anxiety and stress were present between exposure groups, but these were adjusted for in the analysis. Nonetheless, some residual confounding may still have occurred, but the neuropsychological domains affected and the pattern of the effects observed was consistent with our previous study and other international studies of comparable workers, suggesting results are robust. Also, additional sensitivity analyses excluding Māori or Pacific people, who performed lower on some tests, had little effect on the results (S9 Table). Another limitation was the high correlation between age and duration of employment (Spearmans coefficient, 0.91). However, trends observed in analyses adjusted for age were very similar to analyses not adjusting for age (S2 Table) suggesting employment duration is, at least to some degree, independently associated with performance. The limited time available for testing (30–40 minutes) only allowed for a brief test battery, but the tests applied covered functionality previously reported to be affected in solvent exposed workers,[12] and the trends observed were consistent with international studies which employed more comprehensive test batteries.[8, 48] Finally, the sample size was relatively small which prevented more detailed subgroup analyses.

In conclusion, consistent with our previous questionnaire survey in a larger sample of workers, this study has reported significant deficits in objectively measured cognitive performance in solvent- exposed collision repair workers. Analyses focussing on those with the poorest performance suggest that effects in some workers may be relatively severe, despite current airborne exposures in New Zealand being well below international exposure standards.

## Supporting information

S1 Table Neuropsychological test scores based on the lowest 5^th^, 10^th^ and 20^th^ percentiles for comparison and collision repair workers—Excluding Māori and Pacific persons. (DOCX)

S2 Table Neuropsychological test scores for collision repair workers stratified by employment duration—Excluding age from the regression model. (DOCX)

S3 Table Analyses adjusted for lifetime alcohol (mean drinks per week) in place of alcohol consumption in the past 48 hours. (DOCX)

S4 Table Analyses adjusted for lifetime alcohol (frequency) in place of alcohol consumption in the past 48 hours. (DOCX)

S5 Table Neuropsychological test scores based on the lowest 5^th^, 10^th^ and 20^th^ percentiles for collision repair workers stratified by employment duration. (DOCX)

S6 Table Neuropsychological test scores for collision repair workers tested at the start of the week (Monday-Wednesday) and the end of the week (Thursday-Friday). (DOCX)

S7 Table Neuropsychological test scores for comparison and collision repair workers—Excluding reference workers who reported exposure to solvents (n = 7). (DOCX)

S8 Table Neuropsychological test scores for comparison and collision repair workers—Excluding current office workers (n = 4). (DOCX)

S9 Table Neuropsychological test scores for comparison and collision repair workers—Excluding Māori and Pacific persons. (DOCX)

S10 Table Neuropsychological test scores for comparison and collision repair workers—Adjusted for both alcohol consumption in the past 48 hours and lifetime alcohol (mean drinks per week). (DOCX)

S11 Table Characteristics of study populations—Comparison of demographic characteristics of current study and previous study participants. (DOCX)

S1 File Dataset including data points behind means, medians and variance measures. (XLSX)
