# Supplementary material for: Neuropsychological performance in solvent-exposed vehicle collision repair workers in New Zealand
Source: PLoS One. 2017 Dec 13;12(12):e0189108. doi: 10.1371/journal.pone.0189108 (PMC5728539; doi:10.1371/journal.pone.0189108)
Supplement: S1 Table — (DOCX) [file pone.0189108.s001.docx]

**S1 table – Neuropsychological test scores based on the lowest 5^th^, 10^th^ and 20^th^ percentiles for**

**comparison and collision repair workers – excluding Māori and Pacific persons.**

|  | **Reference Group** | **All Collision repair** | | |
| --- | --- | --- | --- | --- |
|  | **(n=30)** | **(n=42)** | | |
|  |  |  | **Unadjusted** | **Adjusted** |
| **RBANS battery** | **N (%)** | **N (%)** | **OR (95%CI)** | **OR (95%CI)** |
| ***Immediate memory*** |  |  |  |  |
| 5th percentile | 0 (0.0) | 3 (7.1) | - | - |
| 10th percentile | 0 (0.0) | 8 (19.1) | - | - |
| 20th percentile | 8 (26.7) | 18 (42.9) | 2.1 (0.7-5.7) | 2.6 (0.6-10.7) |
| ***Visuospatial/Construction*** |  |  |  |  |
| 5th percentile | 1 (3.3) | 0 (0.0) | - | - |
| 10th percentile | 2 (6.7) | 2 (4.7) | 0.7 (0.1-5.3) | 1.8 (0.0-70.4) |
| 20th percentile | 6 (20.0) | 10.0 (23.8) | 1.3 (0.4-3.9) | 1.7 (0.4-6.9) |
| ***Language*** |  |  |  |  |
| h5th percentile | 0 (0.0) | 1 (2.4) | - | - |
| 10th percentile | 0 (0.0) | 2 (4.8) | - | - |
| 20th percentile | 1 (3.3) | 7 (16.7) | 5.8 (0.7-49.9) | **8.2 (0.7-95.2)^** |
| ***Attention*** |  |  |  |  |
| 5th percentile | 1 (3.3) | 7 (16.7) | 5.8 (0.7-49.9) | - |
| 10th percentile | 2 (6.7) | 11 (26.2) | **5.0 (1.0-24.4)*** | **10.9 (1.1-104.0)*** |
| 20^th^ percentile | 5 (16.7) | 17 (40.5) | **3.4 (1.1-10.6)*** | 2.9 (0.8-10.9) |
| ***Delayed Memory*** |  |  |  |  |
| 5^th^ percentile | 1 (3.3) | 2 (4.8) | 1.5 (0.1-16.8) | - |
| 10^th^ percentile | 2 (6.7) | 2 (4.8) | 0.7 (0.1-5.3) | - |
| 20^th^ percentile | 4 (13.3) | 9 (21.4) | 1.8 (0.5-6.4) | 1.6 (0.4-7.1) |
| ***RBANS total scale*** |  |  |  |  |
| 5^th^ percentile | 0 (0.0) | 1 (2.4) |  | - |
| 10^th^ percentile | 0 (0.0) | 3 (7.1) | - | - |
| 20^th^ percentile | 3 (10.0) | 12 (28.6) | **3.6 (0.9-14.1)^** | **6.1 (0.9-40.4)^** |

^ = p<0.1,* = p<0.05, ** = p<0.01

Adjusted for alcohol consumption in the past 48 hours, smoking status, DASS A, S and D, test time (of day)

and test day (of week) and premorbid intelligence (NART).
