# Supplementary material for: Neuropsychological performance in solvent-exposed vehicle collision repair workers in New Zealand
Source: PLoS One. 2017 Dec 13;12(12):e0189108. doi: 10.1371/journal.pone.0189108 (PMC5728539; doi:10.1371/journal.pone.0189108)
Supplement: S2 Table — (DOCX) [file pone.0189108.s002.docx]

**S2 Table – Neuropsychological test scores for collision repair workers stratified by employment duration – excluding age from the**

**regression model**

|  |  | **Employment Duration (mean years)** | | | |
| --- | --- | --- | --- | --- | --- |
|  | **Reference Group** | **< 17 years (10.5)** | | **>17 years (28.4)** | |
|  | **(n=51)** | **(N = 23)** | | **(n = 24)** | |
| ***Immediate memory*** | **Mean (SD)** | **Mean (SD)** | **Difference (95% CI)** | **Mean (SD)** | **Difference (95% CI)** |
| RBANS 1 (list learning) | 29.6 (4.0 ) | 30.0 (4.6) | -0.1 (-2.7, 2.5) | 26.5 (3.6) | **-4.0 (-6.5, -1.4)**** |
| RBANS 2 (story memory) | 16.9 (3.6 ) | 15.6 (4.4) | -0.6 (-3.1, 2.0) | 15.6 (3.1) | -1.4 (-3.9, 1.1) |
| ***Visuospatial/Construction*** |  |  |  |  |  |
| RBANS 3 (figure copy) | 17.1 (2.5) | 18.1 (1.5) | 0.5 (-1.1, 2.1) | 17.5 (2.0) | 0.1 (-1.5, 1.6) |
| RBANS 4 (line orientation) | 18.8 (1.9) | 19.0 (2.0) | 0.9 (-0.6, 2.3) | 18.3 (2.1) | 0.1 (-1.3, 1.5) |
| ***Language*** |  |  |  |  |  |
| RBANS 5 (picture naming) | 9.5 (2.0) | 10.0 (0.0) | 0.4 (-0.6, 1.4) | 10.0 (0.0) | 0.1 (-0.9, 1.0) |
| RBANS 6 (semantic fluency) | 21.5 (5.2) | 21.2 (4.4) | -1.4 (-4.6, 1.9) | 20.9 (3.3) | -2.2 (-5.3, 1.0) |
| ***Attention*** |  |  |  |  |  |
| RBANS 7a (digit span forward) | 10.5 (2.3) | 10.1 (2.2) | **-1.4 (-3.0, 0.2)^** | 10.9 (2.7) | -1.0 (-2.5, 0.6) |
| RBANS 7b (digit span backward) | 7.8 (2.3) | 6.3 (2.0) | **-1.6 (-3.2, 0.0)*** | 6.2 (2.1) | **-2.1 (-3.6, -0.5)**** |
| RBANS 7c (digit span total) | 18.2 (4.1) | 16.4 (3.5) | **-2.9 (-5.6, -0.3)*** | 17.1 (4.2) | **-3.0 (-5.6, -0.4)*** |
| RBANS 8 (coding) | 50.6 (9.4) | 46.6 (7.9) | **-8.3 (-14.7, -1.9)*** | 45.1 (9.8) | **-7.7 (-13.9, -1.5)*** |
| ***Delayed Memory*** |  |  |  |  |  |
| RBANS 9 (list recall) | 7.0 (1.7) | 6.8 (1.6) | 0.4 (-0.8, 1.5) | 4.1 (2.0) | **-2.3 (-3.4, -1.2)**** |
| RBANS 10 (list recognition) | 19.6 (1.7) | 19.8 (0.4) | 0.2 (-0.7, 1.2) | 19.4 (0.7) | -0.3 (-1.2, 0.7) |
| RBANS 11 (story recall) | 9.2 (2.2) | 9 (2.3) | 0.8 (-0.7, 2.4) | 7.2 (2.5) | **-1.5 (-3.0, 0.0)^** |
| RBANS 12 (figure recall) | 14.2 (3.4) | 15.1 (3.0) | 1.0 (-1.3, 3.3) | 12.4 (2.8) | -1.5 (-3.8, 0.7) |
| **Additional Tests** |  |  |  |  |  |
| ***Visual Attention/Reaction Time*** |  |  |  |  |  |
| Trails Aˠ | 23.8 (9.9) | 23.3 (6.7) | -4.8 (- 10.9, 1.3) | 25.1 (6.9) | **-7.0 (- 12.9, -1.1)*** |
| Trails Bˠ | 68.1 (29.1) | 71.2 (27.4) | -4.7 (- 23.5, 14.2) | 77.4 (30.2) | -9.8 (- 28.1, 8.4) |
| Stroop (I) | 2.0 (10.7) | 2.4 (7.3) | -1.6 (-8.5, 5.4) | -2.4 (7.2) | **-7.3 (-14.0, -0.6)*** |
| ***Motor speed/Dexterity*** |  |  |  |  |  |
| coin rot. Dominant hand | 33.7 (5.3) | 31.9 (6.4) | - | 32.7 (6.1) | 1.0 (-2.4, 4.4) |
| coin rot. Non-dominant hand | 31.3 (5.2) | 27.7 (6.5) | **-5.6 (-9.6, -1.7)**** | 28.9 (4.8) | **-3.4 (-7.2, 0.4)^** |

^ = p<0.1,* = p<0.05, ** = p<0.01

Adjusted for ethnicity, job title, alcohol consumption in the past 48 hours, smoking status, DASS A, S and D,

test time (of day) and test day (of week), symptom validity/malingering and premorbid intelligence (NART).

ˠTrails A and B - time to complete each test, therefore higher score represents poorer performance on test –

Algebraic sign of coefficient changed accordingly
