# Supplementary material for: Neuropsychological performance in solvent-exposed vehicle collision repair workers in New Zealand
Source: PLoS One. 2017 Dec 13;12(12):e0189108. doi: 10.1371/journal.pone.0189108 (PMC5728539; doi:10.1371/journal.pone.0189108)
Supplement: S3 Table — (DOCX) [file pone.0189108.s003.docx]

**Table A. Neuropsychological test scores for Comparison and collision repair workers – adjusted for lifetime alcohol (mean drinks per week) in place of alcohol consumption in the past 48 hours.**

|  | **Comparison group** | **All Collision repair** | | **Panel Beaters** | | **Spray painters** | |
| --- | --- | --- | --- | --- | --- | --- | --- |
| **RBANS battery** | **(n=51)** | **(n=47)** | | **(n= 13)** | | **(n=34)** | |
| ***Immediate memory*** | **Mean (SD)** | **Mean (SD)** | **Difference (95%CI)** | **Mean (SD)** | **Difference (95%CI)** | **Mean (SD)** | **Difference (95%CI)** |
| RBANS 1 (list learning) | 29.6 (4.0 ) | 28.4 (4.5) | -0.9 (-2.5, 0.6) | 27.4 (4.6) | **-2.1 (-4.3, 0.2)^** | 28.8 (4.4) | -0.4 (-2.1, 1.3) |
| RBANS 2 (story memory) | 16.9 (3.6 ) | 15.7 (3.9) | -1.2 (-2.7, 0.3) | 16.7 (3.1) | -0.9 (-3.1, 1.3) | 15.3 (4.2) | **-1.3 (-3.0, 0.4)^** |
| Total scale Immediate Memory | 95.6 (12.6) | 91.9 (13.9) | -2.6 (-7.9, 2.7) | 93.5 (9.3) | -5.2 (-12.8, 2.5) | 91.3 (15.3) | -1.6 (-7.3, 4.2) |
| ***Visuospatial/Construction*** |  |  |  |  |  |  |  |
| RBANS 3 (figure copy) | 17.1 (2.5) | 17.8 (1.8) | 0.6 (-0.3, 1.6) | 17.5 (1.7) | 0.3 (-1.0, 1.7) | 17.9 (1.9) | 0.8 (-0.3, 1.8) |
| RBANS 4 (line orientation) | 18.8 (1.9) | 18.7 (2.1) | -0.1 (-1.0, 0.8) | 19.3 (1.44) | 0.4 (-0.8, 1.7) | 18.4 (2.3) | -0.4 (-1.4, 0.6) |
| Total scale vis./const. | 99.6 (15.8) | 99.7 (15.2) | -1.9 (-8.5, 4.8) | 100.2 (16.0) | -3.5 (-13.1, 6.2) | 99.5 (15.1) | -1.2 (-8.5, 6.1) |
| ***Language*** |  |  |  |  |  |  |  |
| RBANS 5 (picture naming) | 9.5 (2.0) | 10.0 (0.0) | 0.2 (-0.4, 0.8) | 10.0 (0.0) | 0.3 (-0.6, 1.1) | 10.0 (0.0) | 0.1 (-0.6, 0.8) |
| RBANS 6 (semantic fluency) | 21.5 (5.2) | 21 (3.9) | -1.0 (-3.1, 1.0) | 20.5 (3.7) | -1.7 (-4.5, 1.2) | 21.2 (4.0) | -0.8 (-3.0, 1.4) |
| Total scale Language | 98.1 (15.0) | 97.2 (12.0) | -3.1 (-9.2, 2.9) | 97.1 (8.1) | -3.4 (-12.2, 5.4) | 97.2 (13.3) | -3.0 (-9.7, 3.6) |
| ***Attention*** |  |  |  |  |  |  |  |
| RBANS 7a (digit span forward) | 10.5 (2.3) | 10.3 (2.4) | -0.2 (-1.2, 0.8) | 9.9 (2.3) | -1.1 (-2.5, 0.3) | 10.5 (2.5) | 0.2 (-0.9, 1.3) |
| RBANS 7b (digit span backward) | 7.8 (2.3) | 6.1 (2.0) | **-1.5 (-2.5, -0.5)**** | 6.1 (1.6) | **-1.8 (-3.2, -0.4)*** | 6.1 (2.2) | **-1.3 (-2.4, -0.2)*** |
| RBANS 7c (digit span total) | 18.2 (4.1) | 16.5 (3.7) | **-1.7 (-3.3, 0.0)*** | 16.0 (2.7) | **-2.9 (-5.3, -0.5)*** | 16.7 (4.1) | -1.1 (-2.9, 0.7) |
| RBANS 8 (coding) | 50.6 (9.4) | 46.1 (8.4) | **-6.2 (-10, -2.3)**** | 45.0 (6.9) | **-7.6 (-13.1, -2)*** | 46.5 (8.9) | **-5.6 (-9.8, -1.3)*** |
| Total scale Attention | 94.6 (14.2) | 88.6 (16.2) | **-9.5 (-16, -2.9)**** | 87.7 (11.4) | **-13.0 (-22.4, -3.6)**** | 88.9 (17.8) | **-8.0 (-15.1, -0.8)*** |
| ***Delayed Memory*** |  |  |  |  |  |  |  |
| RBANS 9 (list recall) | 7.0 (1.7) | 5.7 (2.2) | **-1.0 (-1.7, -0.2)**** | 5.4 (2.3) | **-1.0 (-2.1, 0.1)^** | 5.8 (2.2) | **-1.0 (-1.8, -0.1)*** |
| RBANS 10 (list recognition) | 19.6 (1.7) | 19.6 (0.6) | 0.0 (-0.6, 0.6) | 19.6 (0.7) | 0.0 (-0.9, 0.8) | 19.6 (0.6) | 0.0 (-0.7, 0.7) |
| RBANS 11 (story recall) | 9.2 (2.2) | 8.4 (2.5) | -0.6 (-1.5, 0.3) | 8.8 (2.4) | -0.2 (-1.5, 1.0) | 8.2 (2.5) | -0.7 (-1.7, 0.3) |
| RBANS 12 (figure recall) | 14.2 (3.4) | 13.9 (3.1) | 0.1 (-1.3, 1.6) | 13.4 (3.3) | -0.2 (-2.3, 1.8) | 14.0 (3.1) | 0.3 (-1.3, 1.9) |
| Total scale Delayed Memory | 96.8 (8.4) | 93.3 (8.5) | -1.4 (-5.4, 2.5) | 94.3 (8.1) | -1.6 (-7.3, 4.1) | 92.9 (8.7) | -1.3 (-5.7, 3.0) |
| ***RBANS total scale*** | 96.4 (10.1) | 92.0 (10.5) | **-5.2 (-9.2, -1.3)*** | 91.9 (7.1) | **-7.8 (-13.5, -2.2)**** | 92.0 (11.7) | **-4.1 (-8.4, 0.1)^** |
| **Additional Tests** |  |  |  |  |  |  |  |
| ***Visual Attention/Reaction Time*** |  |  |  |  |  |  |  |
| Trails Aˠ | 23.8 (9.9) | 24.4 (6.9) | -1.8 (-1.8, 5.3) | 28.15 (8.1) | **-5.5 (-10.5, -0.5)** | 22.9 (5.8) | -0.1 (-3.9, 3.8) |
| Trails Bˠ | 68.1 (29.1) | 73.4 (27.7) | **-11.0 (-22.0, -0.1)*** | 67.4 (22.1) | -6.4 (-9.3, 22.1) | 75.8 (29.5) | **-13.1 (-25.3, -1.0)*** |
| Stroop (I) | 2.0 (10.7) | 0.5 (7.3) | -3.3 (-7.6, 1.0) | -1.5 (6.7) | -4.6 (-10.7, 1.6) | 1.2 (7.5) | -2.8 (-7.5, 2.0) |
| ***Motor speed/Dexterity*** |  |  |  |  |  |  |  |
| Coin rot. Dominant hand | 33.7 (5.3) | 31.9 (6.2) | **-2.8 (-5.3, -0.4)*** | 30.00 (6.3) | -4.0 (-7.5, -0.5) | 32.6 (6.1) | **-2.3 (-5.0, 0.4)^** |
| Coin rot. Non-dominant | 31.3 (5.2) | 28.2 (5.7) | **-3.2 (-5.6, -0.7)*** | 26.69 (5.0) | -4.3 (-7.8, -0.8) | 28.8 (5.8) | **-2.6 (-5.3, 0.1)^** |

^ = p<0.1,* = p<0.05, ** = p<0.01

Adjusted for age, ethnicity, Lifetime Alcohol (Mean drinks p/week**)**, smoking status, DASS A, S and D, test time (of day) and test day (of week), symptom validity/malingering (Rey 15 item) and premorbid intelligence (NART).

ˠTrails A and B - time to complete each test, therefore higher score represents poorer performance on test - Algebraic sign of coefficient changed accordingly

**Table B. Neuropsychological test scores based on the bottom 5^th^, 10^th^ and 20^th^ percentiles for Comparison and collision repair workers – adjusted for lifetime alcohol (mean drinks per week) in place of alcohol consumption in the past 48 hours.**

|  | **Comparison group** | **All Collision repair** | | |
| --- | --- | --- | --- | --- |
|  | **(n=51)** | **(n=47)** | | |
|  |  |  | **Unadjusted** | **Adjusted** |
| **RBANS battery** | **N (%)** | **N (%)** | **OR (95%CI)** | **OR (95%CI)** |
| ***Immediate memory*** |  |  |  |  |
| 5th percentile | 2 (3.9) | 4 (8.5) | 2.3 (0.4, 13.1) | - |
| 10th percentile | 4 (7.8) | 10 (21.3) | **3.2 (0.9, 10.9)^** | **10.6 (1.3, 89.6)*** |
| 20th percentile | 17 (33.3) | 21 (44.7) | 1.6 (0.7, 3.7) | 2.1 (0.6, 6.9) |
| ***Visuospatial/Construction*** |  |  |  |  |
| 5th percentile | 4 (7.8) | 1 (2.1) | 0.3 (0.0, 2.4) |  |
| 10th percentile | 5 (9.8) | 4 (8.5) | 0.9 (0.2, 3.4) | 4.0 (0.3, 61.8) |
| 20th percentile | 13 (25.5) | 13 (27.7) | 1.1 (0.5, 2.7) | 1.9 (0.6, 6.2) |
| ***Language*** |  |  |  |  |
| 5th percentile | 3 (5.9) | 1 (2.1) | 0.3 (0.0, 3.5) | - |
| 10th percentile | 5 (9.8) | 3 (6.4) | 0.6 (0.1, 2.8) | 1.5 (0.1, 15.1) |
| 20th percentile | 7 (13.7) | 9 (19.2) | 1.5 (0.5, 4.4) | **4.3 (0.9, 20.5)^** |
| ***Attention*** |  |  |  |  |
| 5th percentile | 4 (7.8) | 11 (23.4) | **3.6 (1.1, 12.2)*** | **19.0 (1.4, 252.6)*** |
| 10th percentile | 8 (15.7) | 15 (31.9) | **2.5 (1.0, 6.7)^** | **8.6 (1.7, 44.9)*** |
| 20th percentile | 12 (23.5) | 22 (46.8) | **2.9 (1.2, 6.8)*** | **5.2 (1.5, 17.8)**** |
| ***Delayed Memory*** |  |  |  |  |
| 5th percentile | 1 (2.0) | 2 (4.3) | 2.2 (0.2, 25.3) | - |
| 10th percentile | 2 (3.9) | 2 (4.3) | 1.1 (0.1, 8.1) | 0.0 (0.0, 7.6) |
| 20th percentile | 4 (7.8) | 11 (23.4) | **3.6 (1.1, 12.2)*** | 3.1 (0.8, 12.3) |
| ***RBANS total scale*** |  |  |  |  |
| 5th percentile | 2 (3.9) | 1 (2.1) | 0.5 (0.0, 6.1) | - |
| 10th percentile | 5 (9.8) | 5 (10.6) | 1.1 (0.3, 4.1) | 4.1 (0.4, 40.8) |
| 20th percentile | 9 (17.7) | 17 (36.2) | **2.6 (1.0, 6.7)*** | **14.2 (2.4, 84.5)**** |

^ = p<0.1,* = p<0.05, ** = p<0.01

Adjusted for ethnicity, Lifetime Alcohol (Mean drinks per week**)**, smoking status, DASS A, S and D, NART, test time (of day) and test day (of week).

**Table C. Neuropsychological test scores for collision repair workers stratified by employment duration. – adjusted for lifetime alcohol (mean drinks per week) in place of alcohol consumption in the past 48 hours.**

|  |  | **Employment Duration (mean years)** | | | |
| --- | --- | --- | --- | --- | --- |
|  | **Comparison group** | **< 17 years (10.5)** | | **>17 years (28.4)** | |
|  | **(n=51)** | **(N = 23)** | | **(n = 24)** | |
| ***Immediate memory*** | **Mean (SD)** | **Mean (SD)** | **Difference (95%CI)** | **Mean (SD)** | **Difference (95%CI)** |
| RBANS 1 (list learning) | 29.6 (4.0) | 30.8 (4.2) | -0.9 (-3.4, 1.6) | 26.2 (3.5) | **-3.1 (-5.6, -0.6)*** |
| RBANS 2 (story memory) | 16.9 (3.6) | 15.7 (4.4) | -0.9 (-3.5, 1.6) | 15.6 (3.5) | -0.8 (-3.3, 1.7) |
| Total scale Immediate Memory | 95.6 (12.6) | 93.2 (16.8) | -3.5 (-12.2,5.1) | 90.7 (10.6) | -6.5 (-14.9, 1.8) |
| ***Visuospatial/Construction*** |  |  |  |  |  |
| RBANS 3 (figure copy) | 17.1 (2.5) | 18.0 (1.5) | 0.1 (-1.5, 1.6) | 17.6 (2.1) | 0.6 (-1.0, 2.1) |
| RBANS 4 (line orientation) | 18.8 (1.9) | 18.9 (2.2) | 0.6 (-0.9, 2.0) | 18.5 (2.1) | 0.3 (-1.1, 1.7) |
| Total scale vis./const. | 99.6 (15.8) | 97.4 (14.2) | -5.0 (-15.9,5.9) | 101.9 (16.1) | -2.2(-12.7-8.4) |
| ***Language*** |  |  |  |  |  |
| RBANS 5 (picture naming) | 9.5 (2.0) | 10 (0.0) | 0.2 (-0.8, 1.2) | 10.0 (0.0) | 0.3 (-0.7, 1.3) |
| RBANS 6 (semantic fluency) | 21.5 (5.2) | 21.6 (4.4) | -1.7 (-5.0, 1.7) | 20.4 (3.4) | -1.7 (-4.9, 1.6) |
| Total scale Language | 98.1 (15.0) | 99.1 (11.3) | -0.7(-10.6, 9.1) | 93.4 (12.6) | -5.6 (-15.2, 3.9) |
| ***Attention*** |  |  |  |  |  |
| RBANS 7a (digit span forward) | 10.5 (2.3) | 10.2 (2.3) | **-1.5 (-3.1, 0.1)^** | 10.5 (2.5) | -0.8 (-2.4, 0.8) |
| RBANS 7b (digit span backward) | 7.8 (2.3) | 6.4 (2.0) | **-1.8 (-3.4, -0.2)*** | 5.8 (2.0) | **-1.9 (-3.5, -0.3)*** |
| RBANS 7c (digit span total) | 18.2 (4.1) | 16.7 (3.6) | **-3.2 (-5.9, -0.5)*** | 16.3 (3.9) | **-2.6 (-5.3, 0.0)^** |
| RBANS 8 (coding) | 50.6 (9.4) | 46.4 (8.0) | **-9.6 (-15.9, -3.2)**** | 45.8 (8.9) | **-5.8 (-12.0, 0.4)^** |
| Total scale Attention | 94.6 (14.2) | 82.3 (14.2) | **-20.1(-30.2, -10.0)**** | 94.6 (16.0) | -7.1 (-16.9, 2.7) |
| ***Delayed Memory*** |  |  |  |  |  |
| RBANS 9 (list recall) | 7.0 (1.7) | 7.2 (1.3) | 0.0 (-1.2, 1.1) | 4.3 (1.9) | -1.9 (-3.0, -0.8) |
| RBANS 10 (list recognition) | 19.6 (1.7) | 19.8 (0.4) | 0.0 (-1.0, 1.0) | 19.5 (0.7) | -0.1 (-1.1, 0.9) |
| RBANS 11 (story recall) | 9.2 (2.2) | 9.3 (2.2) | 0.4 (-1.1, 1.9) | 7.5 (2.5) | -0.8 (-2.2, 0.6) |
| RBANS 12 (figure recall) | 14.2 (3.4) | 15.4 (2.5) | 0.8 (-1.6, 3.1) | 12.3 (3.0) | -1.1 (-3.4, 1.2) |
| Total scale Delayed Memory | 96.8 (8.4) | 95.5 (6.3) | 1.0 (-5.4, 7.3) | 91.1 (9.8) | -3.7 (-9.8, 2.5) |
| RBANS total scale^ | 96.4 (10.1) | 91.0 (11.5) | **-8.6 (-15, -2.2)**** | 92.9 (9.7) | **-7.2 (-13.3, -1.0)*** |
| **Additional Tests** |  |  |  |  |  |
| ***Visual Attention/Reaction Time*** |  |  |  |  |  |
| Trails Aˠ | 23.8 (9.9) | 22.8 (6.6) | -6.7 (-12.5, 1.0) | 25.8 (6.9) | 4.4 (-10.0, 1.2) |
| Trails Bˠ | 68.1 (29.1) | 71.2 (27.9) | -11.4 (-29.4, 6.7) | 75.6 (27.9) | 2.0 (-19.5, 15.5) |
| Stroop (I) | 2.0 (10.7) | 3.0 (7.4) | -2.5 (-9.6, 4.5) | -1.9 (6.6) | **-6.3 (-13.2, 0.5)^** |
| ***Motor speed/Dexterity*** |  |  |  |  |  |
| coin rot. Dominant hand | 33.7 (5.3) | 32.0 (6.7) | - | 31.9 (5.8) | **3.2 (-0.5, 6.9)^** |
| coin rot. Non-dominant hand | 31.3 (5.2) | 27.6 (6.7) | **-6.6 (-10.5, -2.6)**** | 28.8 (4.5) | -2.4 (-6.2, 1.5) |

^ = p<0.1,* = p<0.05, ** = p<0.01

Adjusted for age, ethnicity, Job title (spray painter/panel beater), Lifetime Alcohol (Mean drinks p/week), smoking status, DASS A, S and D, test time (of day) and test day (of week), malingering/symptom validity (Rey 15 item) and premorbid intelligence (NART).

ˠTrails A and B - time to complete each test, therefore higher score represents poorer performance on test - Algebraic sign of coefficient changed accordingly
