# Supplementary material for: Neuropsychological performance in solvent-exposed vehicle collision repair workers in New Zealand
Source: PLoS One. 2017 Dec 13;12(12):e0189108. doi: 10.1371/journal.pone.0189108 (PMC5728539; doi:10.1371/journal.pone.0189108)
Supplement: S5 Table — (DOCX) [file pone.0189108.s005.docx]

**S5 table - Neuropsychological test scores based on the lowest 5^th^, 10^th^ and 20^th^ percentiles for collision repair workers stratified by employment duration**

|  | **Reference Group** | **< 17 years (10.5)** | | **>17 years (28.4)** | |
| --- | --- | --- | --- | --- | --- |
|  | **(n=51)** | **(N = 23)** | | **(n = 24)** | |
| **RBANS battery** | **N (%)** | **N (%)** | **OR (95%CI)** | **N (%)** | **OR (95%CI)** |
| ***Immediate memory*** |  |  |  |  |  |
| 5th percentile | 2 (3.9) | 2 (8.7) | - | 2 (8.3) | - |
| 10th percentile | 4 (7.8) | 6 (26.9) | **13.0 (1.5-117.2)*** | 4 (16.7) | **8.8 (0.7-107.7)^** |
| 20th percentile | 17 (33.3) | 11 (57.8) | 2.0 (0.5-9.0) | 10 (41.7) | 2.0 (0.5-8.6) |
| ***Visuospatial/Construction*** |  |  |  |  |  |
| 5th percentile | 4 (7.8) | 1 (4.3) | - | 0 (0.0) | - |
| 10th percentile | 5 (9.8) | 3 (13.0) | 13.3 (0.3-569.0) | 1 (4.2) | 2.1 (0-142.6) |
| 20th percentile | 13 (25.5) | 6 (26.1) | 1.6 (0.4-6.3) | 7 (29.2) | 2.4 (0.6-9.6) |
| ***Language*** |  |  |  |  |  |
| 5th percentile | 3 (5.9) | 0 (0.0) | - | 1 (4.2) | - |
| 10th percentile | 5 (9.8) | 2 (8.7) | 7.5 (0.2-364.1) | 1 (4.2) | 3.5 (0.1-238.5) |
| 20th percentile | 7 (13.7) | 5 (21.7) | **4.7 (0.8-27.8)^** | 4 (16.7) | 4.2 (0.6-27.5) |
| ***Attention*** |  |  |  |  |  |
| 5th percentile | 4 (7.8) | 8 (34.8) | **53.3 (3.3-862.5)**** | 3 (12.5) | 4.6 (0.3-73.9) |
| 10th percentile | 8 (15.7) | 10 (43.5) | **12.5 (2.3-68.6)**** | 5 (20.8) | 4.3 (0.6-28.8) |
| 20th percentile | 12 (23.5) | 16 (69.6) | **21.1 (4.2-105.9)**** | **6 (25.0)** | 1.2 (0.3-5.5) |
| ***Delayed Memory*** |  |  |  |  |  |
| 5th percentile | 1 (2.0) | 0 (0.0) | - | 2 (8.3) | - |
| 10th percentile | 2 (3.9) | 0 (0.0) | - | 2 (8.3) | - |
| 20th percentile | 4 (7.8) | 3 (13.0) | 1.3 (0.2-8.4) | 8 (33.3) | **4.8 (1-21.9)*** |
| ***RBANS total scale*** |  |  |  |  |  |
| 5th percentile | 2 (3.9) | 1 (4.4) | - | 0 (0.0) | - |
| 10th percentile | 5 (9.8) | 4 (17.4) | **16.4 (0.7-387.6)^** | 1 (4.2) | 1.8 (0.1-53.5) |
| 20th percentile | 9 (17.7) | 8 (34.8) | **10.6 (1.5-73.4)*** | 9 (37.5) | **19.7 (2.5-152.7)**** |

^ = p<0.1,* = p<0.05, ** = p<0.01

Adjusted for ethnicity, alcohol consumption in the past 48 hours, smoking status, DASS A, S and D, test time (of day) and

test day (of week) and premorbid intelligence (NART).
