# Supplementary material for: Neuropsychological performance in solvent-exposed vehicle collision repair workers in New Zealand
Source: PLoS One. 2017 Dec 13;12(12):e0189108. doi: 10.1371/journal.pone.0189108 (PMC5728539; doi:10.1371/journal.pone.0189108)
Supplement: S6 Table — (DOCX) [file pone.0189108.s006.docx]

**S6 Table - Neuropsychological test scores for collision repair workers tested at the start of the week (Monday-Wednesday) and the end of the week (Thursday-Friday).**

|  | **Reference group** | **All Collision repair workers** | | | |
| --- | --- | --- | --- | --- | --- |
|  | **n=51** | **Early week (n=38)** | **Adjusted*** | **Late week (n=13)** | **Adjusted*** |
| **RBANS battery** | **N (%)** | **N (%)** | **OR (95%CI)** | **N (%)** | **OR (95%CI)** |
| ***Immediate memory*** |  |  |  |  |  |
| RBANS 1 (list learning) | 29.6 (4.0) | 28.1 (4.7) | **-2.7 (-5.2, -0.1)*** | 29.3 (3.8) | -0.8 (-4.1, 2.4) |
| RBANS 2 (story memory) | 16.9 (3.6) | 16.4 (3.7) | -0.5 (-2.8, 1.8) | 13.8 (3.9) | **-2.6 (-5.5, 0.3)^** |
| Total scale Immediate Memory | 95.6 (12.6) | **92.4 (13.3)** | -5.5 (-13.4, 2.3) | **90.8 (15.9)** | -3.4 (-13.6, 6.6) |
| ***Visuospatial/Construction*** |  |  |  |  |  |
| RBANS 3 (figure copy) | 17.1 (2.5) | 17.9 (1.8) | 0.5 (-1.0, 1.9) | 17.5 (2.0) | -0.2 (-2.0, 1.7) |
| RBANS 4 (line orientation) | 18.8 (1.9) | 18.7 (2.2) | 0.6 (-0.8, 1.9) | 18.7 (2.2) | 0.3 (-1.4, 2.0) |
| Total scale vis./const. | 99.6 (15.8) | 100.5 (16.4) | -2.7 (-12.6, 7.2) | 97.5 (11.7) | -5.7 (-18.3, 6.9) |
| ***Language*** |  |  |  |  |  |
| RBANS 5 (picture naming) | 9.5 (2.0) | 10.0 (0.0) | 0.2 (-0.7, 1.2) | 10.0 (0.0) | 0.2 (-1.0, 1.4) |
| RBANS 6 (semantic fluency) | 21.5 (5.2) | 22.2 (3.9) | -2.3 (-5.2, 0.7) | 22.2 (3.9) | -0.4 (-4.1, 3.4) |
| Total scale Language | 98.1 (15.0) | 95.9 (12.7) | -4.8 (-13.3, 3.8) | 100.7 (9.4) | 0.1 (-10.9, 11) |
| ***Attention*** |  |  |  |  |  |
| RBANS 7a (digit span forward) | 10.5 (2.3) | 10.8 (2.2) | -0.8 (-2.2, 0.6) | 9.2 (2.7) | **-2.3 (-4.2, -0.5)*** |
| RBANS 7b (digit span backward) | 7.8 (2.3) | 6.1 (1.7) | **-1.9 (-3.3, -0.5)**** | 6.2 (2.7) | **-1.8 (-3.7, -0.0)*** |
| RBANS 7c (digit span total) | 18.2 (4.1) | 16.9 (3.3) | **-2.6 (-5.0, -0.2)*** | 15.4 (4.6) | **-4.1 (-7.2, -1.1)**** |
| RBANS 8 (coding) | 50.6 (9.4) | 46.2 (7.9) | **-8.3 (-14.2, -2.5)**** | 45.6 (9.9) | **-7.0 (-14.4, -0.4)^** |
| Total scale Attention | 94.6 (14.2) | 90.7 (14.2) | **-12.1 (-21.6, -2.5)**** | 83.0 (20.1) | **-16.8 (-29.1, -4.6)**** |
| ***Delayed Memory*** |  |  |  |  |  |
| RBANS 9 (list recall) | 7.0 (1.70) | 5.5 (2.3) | **-1.2 (-2.5, -0.0)*** | 6.2 (2.0) | -0.6 (-2.2, 0.9) |
| RBANS 10 (list recognition) | 19.6 (1.7) | 19.6 (0.7) | -0.1 (-1.0, 0.8) | 19.8 (0.4) | 0.1 (-1.1, 1.2) |
| RBANS 11 (story recall) | 9.2 (2.2) | 8.8 (2.4) | -0.1 (-1.6, 1.3) | 7.4 (2.6) | -1.4 (-3.3, 0.5) |
| RBANS 12 (figure recall) | 14.2 (3.4) | 13.6 (3.1) | -0.7 (-2.9, 1.5) | 14.5 (3.3) | 0.3 (-2.5, 3.1) |
| Total scale Delayed Memory | 96.8 (8.4) | 92.9 (8.0) | -2.1 (-8.0, 3.8) | 94.2 (10.0) | -0.3 (-7.7, 7.2) |
|  |  |  |  |  |  |
| RBANS total scale | 96.4 (10.1) | 92.5 (10.2) | **-7.9 (-13.7, -2.1)*** | 90.5 (11.6) | **-8.1 (-15.5, -0.7)*** |
| ***Additional Tests*** |  |  |  |  |  |
| ***Visual Attention/Reaction Time*** |  |  |  |  |  |
| Trails Aˠ | 23.8 (7.0) | 24.7 (7.0) | -6.0 (-11.6, 0.5) | 23.5 (9.7) | -5.6 (-12.6, 1.5) |
| Trails Bˠ | 68.1 (29.1) | 74.7 (27.8) | -10.6 (-27.5, 6.4) | 70.2 (28.2) | 2.2 (-19.4, 23.8) |
| Stroop (I) | 2.0 (10.7) | -0.5 (6.9) | -5.3 (-11.7, 1.1) | 3.1 (8.0) | -1.9 (-10.0, 6.2) |
| ***Motor speed/Dexterity*** |  |  |  |  |  |
| Coin rot. Dominant hand | 33.7 (5.3) | 31.5 (6.8) | -1.0 (-4.9, 3.0) | 33.0 (4.3) | - |
| Coin rot. Non-dominant | 31.3 (5.2) | 27.4 (5.7) | **-4.9 (-8.6, -1.2)**** | 30.2 (5.1) | -3.3 (-8.0, 1.3) |

^ = p<0.1,* = p<0.05, ** = p<0.01

Adjusted for age, ethnicity, alcohol consumption in the past 48 hours, smoking status, DASS A, S and D, test time (of day) and test day (of week) and premorbid

intelligence (NART).

ˠTrails A and B - time (in seconds) to complete each test, therefore higher score represents poorer performance on test – Algebraic sign of coefficient

changed accordingly
