# Supplementary material for: Neuropsychological performance in solvent-exposed vehicle collision repair workers in New Zealand
Source: PLoS One. 2017 Dec 13;12(12):e0189108. doi: 10.1371/journal.pone.0189108 (PMC5728539; doi:10.1371/journal.pone.0189108)
Supplement: S7 Table — (DOCX) [file pone.0189108.s007.docx]

**S7 Table – Neuropsychological test scores for Comparison and collision repair workers - excluding reference workers who reported exposure to solvents (n=7).**

|  | **Reference Group** | **All Collision repair** | |
| --- | --- | --- | --- |
| **RBANS battery** | **(n=44)** | **n=47** | |
| ***Immediate memory*** | **Mean (SD)** | **Mean (SD)** | **Difference (95% CI)** |
| RBANS 1 (list learning) | 29.7 (4.1) | 28.4 (4.5) | -1.2 (-2.9, 0.5) |
| RBANS 2 (story memory) | 16.8 (3.7) | 15.7 (3.9) | -1.1 (-2.7, 0.6) |
| Total scale Immediate Memory | 95.5 (12.6) | 91.9 (13.9) | -2.8 (-8.5, 2.8) |
| ***Visuospatial/Construction*** |  |  |  |
| RBANS 3 (figure copy) | 17.0 (2.6) | 17.8 (1.8) | 0.6 (-0.4, 1.6) |
| RBANS 4 (line orientation) | 18.7 (1.9) | 18.7 (2.1) | -0.2 (-1.1 , 0.8) |
| Total scale vis./const. | 99.7 (16.0) | 99.7 (15.2) | -3.5 (-10.4, 3.4) |
| ***Language*** |  |  |  |
| RBANS 5 (picture naming) | 9.5 (2.1) | 10.0 (0.0) | 0.2 (-0.5, 0.9) |
| RBANS 6 (semantic fluency) | 21.2 (5.1) | 21.2 (3.9) | -0.8 (-3.0, 1.3) |
| Total scale Language | 97.2 (15.4) | 97.2 (12.0) | -1.7 (-8.1, 4.6) |
| ***Attention*** |  |  |  |
| RBANS 7a (digit span forward) | 10.3 (2.2) | 10.3 (2.4) | 0.0 (-1.1, 1.0) |
| RBANS 7b (digit span backward) | 7.7 (2.3) | 6.1 (2.0) | **-1.3 (-2.4, -0.3)*** |
| RBANS 7c (digit span total) | 17.9 (3.9) | 16.5 (3.7) | -1.3 (-3.1, 0.5) |
| RBANS 8 (coding) | 49.7 (8.9) | 46.1 (8.4) | **-5.0 (-9.1, -1.0)*** |
| Total scale Attention | 93.8 (13.8) | 88.6 (16.2) | **-9.1 (-15.9, -2.3)**** |
| ***Delayed Memory*** |  |  |  |
| RBANS 9 (list recall) | 6.9 (1.7) | 5.7 (2.2) | **-0.9 (-1.7, -0.1)*** |
| RBANS 10 (list recognition) | 19.6 (1.8) | 19.6 (0.6) | 0.0 (-0.7, 0.7) |
| RBANS 11 (story recall) | 9.2 (2.3) | 8.4 (2.5) | -0.7 (-1.7, 0.3) |
| RBANS 12 (figure recall) | 14.1 (3.5) | 13.9 (3.1) | 0.1 (-1.5, 1.6) |
| Total scale Delayed Memory | 96.5 (8.8) | 93.3 (8.5) | -0.9 (-5.3, 3.5) |
|  |  |  |  |
| RBANS total scale | 96.1 (10.1) | 92.0 (10.5) | **-5.5 (-9.7, -1.4)**** |
| **Additional Tests** |  |  |  |
| ***Visual Attention/Reaction Time*** |  |  |  |
| Trails Aˠ | 24.4 (10.5) | 24.4 (6.9) | -1.6 (- 5.6, 2.4) |
| Trails Bˠ | 69.6 (30.3) | 73.4 (27.7) | **10.4 (- 22.2, -1.4)^** |
| Stroop (I) | 1.8 (10.9) | 0.5 (7.3) | -3.3 (-7.9, 1.3) |
| ***Motor speed/Dexterity*** |  |  |  |
| Coin rot. Dominant hand | 33.1 (5.5) | 31.9 (6.2) | **-2.5 (-5.3, 0.2)^** |
| Coin rot. Non-dominant | 31.2 (5.5) | 28.2 (5.7) | **-3.2 (-6.0, -0.5)*** |

^ = p<0.1,* = p<0.05, ** = p<0.01

Adjusted for age, ethnicity, alcohol consumption in the past 48 hours, smoking status,

DASS A, S and D, test time (of day) and test day (of week), symptom validity/malingering and premorbid intelligence (NART).

ˠTrails A and B - time to complete each test, therefore higher score represents poorer performance on test - Algebraic sign of coefficient changed accordingly
