# Supplementary material for: Neuropsychological performance in solvent-exposed vehicle collision repair workers in New Zealand
Source: PLoS One. 2017 Dec 13;12(12):e0189108. doi: 10.1371/journal.pone.0189108 (PMC5728539; doi:10.1371/journal.pone.0189108)
Supplement: S9 Table — (DOCX) [file pone.0189108.s009.docx]

**S9 Table. Neuropsychological test scores for Comparison and collision repair workers –**

**excluding Māori and Pacific persons.**

|  | **Reference Group** | **All Collision repair** | |
| --- | --- | --- | --- |
| **RBANS battery** | **(n=30)** | **(n=42)** | |
| ***Immediate memory*** | **Mean (SD)** | **Mean (SD)** | **Difference (95% CI)** |
| RBANS 1 (list learning) | 29.9 (3.3) | 28.4 (4.6) | -1.2 (-3.2, 0.7) |
| RBANS 2 (story memory) | 17.6 (3.1) | 16.0 (3.9) | -1.0 (-2.8, 0.8) |
| Total scale Immediate Memory | 98.1 (12.0) | 92.8 (14.0) | -2.6 (-9.0, 3.9) |
| ***Visuospatial/Construction*** |  |  |  |
| RBANS 3 (figure copy) | 17.1 (2.4) | 17.9 (1.8) | 0.6 (-0.5, 1.7) |
| RBANS 4 (line orientation) | 18.8 (2.1) | 19.0 (1.7) | 0.6 (-0.5, 1.6) |
| Total scale vis./const. | 101.7 (14.9) | 101.2 (14.2) | -1.4 (-8.9, 6.1) |
| ***Language*** |  |  |  |
| RBANS 5 (picture naming) | 10.0 (0.2) | 10.0 (0.0) | 0.0 (0.0, 0.1) |
| RBANS 6 (semantic fluency) | 22.3 (4.8) | 21.4 (3.9) | -1.1(-3.1, 1.0) |
| Total scale Language | 101.5 (9.9) | 97.9 (12.2) | -2.2 (-7.3, 3.0) |
| ***Attention*** |  |  |  |
| RBANS 7a (digit span forward) | 10.7 (2.4) | 10.5 (2.5) | 0.0 (-1.2, 1.3) |
| RBANS 7b (digit span backward) | 8.1 (2.5) | 6.1 (2.0) | **-1.6 (-2.7, -0.4)*** |
| RBANS 7c (digit span total) | 18.7 (4.4) | 16.6 (3.8) | -1.5 (-3.6, 0.6) |
| RBANS 8 (coding) | 52.2 (10.3) | 46.9 (8.2) | **-5.5 (-10.3, -0.8)*** |
| Total scale Attention | 98.8 (13.6) | 90.3 (16.1) | **-8.1 (-16.5, - 0.4)^** |
| ***Delayed Memory*** |  |  |  |
| RBANS 9 (list recall) | 6.7 (1.7) | 5.6 (2.2) | **-1.1 (-2, -0.2)*** |
| RBANS 10 (list recognition) | 19.4 (2.32 | 19.6 (0.6) | 0.0 (-0.8, 0.8) |
| RBANS 11 (story recall) | 9.0 (1.9) | 8.6 (2.5) | -0.1 (-1.1, 1.0) |
| RBANS 12 (figure recall) | 14.0 (2.4) | 13.9 (3.2) | 0.1 (-1.4, 1.6) |
| Total scale Delayed Memory | 95.5 (8.5) | 93.6 (8.8) | -0.2 (-4.9, 4.5) |
|  |  |  |  |
| RBANS total scale | 98.9 (8.2) | 93.3 (10.3) | **-4.7 (-9.4, -0.0)*** |
| **Additional Tests** |  |  |  |
| ***Visual Attention/Reaction Time*** |  |  |  |
| Trails Aˠ | 23.0 (9.1) | 23.4 (5.9) | -0.4 (-4.0, 3.3) |
| Trails Bˠ | 69.7 (29.5) | 70.3 (27.0) | -7.6 (-19.7, 4.4) |
| Stroop (I) | 2.6 (11.8) | 0.9 (7.4) | -2.4 (-7.4, 2.6) |
| ***Motor speed/Dexterity*** |  |  |  |
| Coin rot. Dominant hand | 34.5 (4.5) | 32.8 (5.6) | -2.0 (-4.8, 0.7) |
| Coin rot. Non-dominant | 32.8 (4.5) | 32.8 (5.6) | -2.0 (-4.5, 0.6) |

^ = p<0.1,* = p<0.05, ** = p<0.01

Adjusted for age, alcohol consumption in the past 48 hours, smoking status, DASS A, S and D, test time

(of day) and test day (of week) and premorbid intelligence (NART).

ˠTrails A and B - time to complete each test, therefore higher score represents poorer performance on test –

Algebraic sign of coefficient changed accordingly
