# Supplementary material for: Neuropsychological performance in solvent-exposed vehicle collision repair workers in New Zealand
Source: PLoS One. 2017 Dec 13;12(12):e0189108. doi: 10.1371/journal.pone.0189108 (PMC5728539; doi:10.1371/journal.pone.0189108)
Supplement: S10 Table — (DOCX) [file pone.0189108.s010.docx]

|  | **Reference Group** | **All Collision repair** | | **Panel Beaters** | | **Spray painters** | |
| --- | --- | --- | --- | --- | --- | --- | --- |
| **RBANS battery** | **(n=51)** | **(n=43)** | | **(n= 11)** | | **(n=32)** | |
| ***Immediate memory*** | **Mean (SD)** | **Mean (SD)** | **Difference (95% CI)** | **Mean (SD)** | **Difference (95% CI)** | **Mean (SD)** | **Difference (95% CI)** |
| RBANS 1 (list learning) | 29.6 (4.0 ) | 28.6 (4.5) | -0.9 (-2.4, 0.7) | 28.2 (4.6) | **-2.1 (-4.3, 0.2)^** | 28.8 (4.6) | -0.3 (-2.0, 1.5) |
| RBANS 2 (story memory) | 16.9 (3.6 ) | 15.6 (3.9) | -1.2 (-2.8, 0.3) | 16.2 (3.2) | -0.9 (-3.1, 1.4) | 15.4 (4.2) | **-1.4 (-3.1, 0.3)^** |
| Total scale Immediate Memory | 95.6 (12.6) | 92.2 (14.4) | -2.6 (-7.9, 2.8) | 93.8 (10.1) | -5.2 (-12.9, 2.5) | 91.7 (15.8) | -1.4 (-7.3, 4.4) |
| ***Visuospatial/Construction*** |  |  |  |  |  |  |  |
| RBANS 3 (figure copy) | 17.1 (2.5) | 17.8 (1.7) | 0.6 (-0.4, 1.6) | 17.0 (1.4) | 0.3 (-1.0, 1.7) | 18.1 (1.7) | 0.7 (-0.3, 1.8) |
| RBANS 4 (line orientation) | 18.8 (1.9) | 18.7 (2.1) | -0.2 (-1.1, 0.7) | 19.2 (1.5) | 0.4 (-0.8, 1.7) | 18.5 (2.2) | -0.5 (-1.5, 0.5) |
| Total scale vis./const. | 99.6 (15.8) | 99.3 (14.2) | -2.1 (-8.8, 4.6) | 95.5 (12.2) | -3.5 (-13.2, 6.2) | 100.6 (14.8) | -1.5 (-8.9, 5.9) |
| ***Language*** |  |  |  |  |  |  |  |
| RBANS 5 (picture naming) | 9.5 (2.0) | 10.0 (0.0) | 0.2 (-0.5, 0.8) | 10.0 (0.0) | 0.3 (-0.6, 1.1) | 10.0 (0.0) | 0.1 (-0.6, 0.8) |
| RBANS 6 (semantic fluency) | 21.5 (5.2) | 21.0 (4.0) | -1.0 (-3.0, 1.1) | 20.5 (3.6) | -1.7 (-4.5, 1.2) | 21.3 (4.1) | -0.6 (-2.9, 1.6) |
| Total scale Language | 98.1 (15.0) | 97.2 (12.4) | -2.3 (-8.1, 3.6 | 97.0 (8.3) | -3.3 (-11.8, 5.1) | 97.3 (13.7) | -1.8 (-8.2, 4.6) |
| ***Attention*** |  |  |  |  |  |  |  |
| RBANS 7a (digit span forward) | 10.5 (2.3) | 10.4 (2.5) | -0.2 (-1.2, 0.8) | 10.(2.4) | -1.1 (-2.5, 0.3) | 10.6 (2.5) | 0.2 (-0.9, 1.3) |
| RBANS 7b (digit span backward) | 7.8 (2.3) | 6.3 (2.0) | **-1.5 (-2.5, -0.5)*** | 6.4 (1.5) | **-1.8 (-3.3, -0.4)*** | 6.3 (2.2) | **-1.3 (-2.4, -0.2)*** |
| RBANS 7c (digit span total) | 18.2 (4.1) | 16.7 (3.8) | **-1.7 (-3.3, 0.0)*** | 16.4 (2.7) | **-2.9 (-5.3, -0.5)*** | 16.8 (4.1) | -1.1 (-2.9, 0.8) |
| RBANS 8 (coding) | 50.6 (9.4) | 46.0 (8.6) | **-5.9 (-9.8, -2.0)**** | 44.3 (7.4) | **-7.5 (-13, -2.0)*** | 46.6 (9.0) | **-5.1 (-9.4, -0.8)*** |
| Total scale Attention | 94.6 (14.2) | 88.2 (16.8) | **-9.2 (-15.8, -2.6**)** | 86.3 (11.8) | **-13.0 (-22.4,-3.5)**** | 88.8 (18.3) | **7.6 (-14.8, -0.3)*** |
| ***Delayed Memory*** |  |  |  |  |  |  |  |
| RBANS 9 (list recall) | 7.0 (1.7) | 5.7 (2.2) | **-1.0 (-1.8, -0.3)** | 5.4 (2.3) | **-1.0 (-2.1, 0.1)^** | 5.9 (2.2) | **-1.0 (-1.8, -0.2)*** |
| RBANS 10 (list recognition) | 19.6 (1.7) | 19.7 (0.6) | 0.0 (-0.6, 0.6) | 19.7 (0.5) | 0.0 (-0.9, 0.8) | 19.6 (0.6) | 0.0 (-0.7, 0.6) |
| RBANS 11 (story recall) | 9.2 (2.2) | 8.3 (2.5) | -0.6 (-1.5, 0.3) | 8.5 (2.5) | -0.2 (-1.5, 1.0) | 8.3 (2.6) | -0.8 (-1.8, 0.3) |
| RBANS 12 (figure recall) | 14.2 (3.4) | 14.0 (3.2) | 0.1 (-1.3, 1.6) | 13.7 (3.5) | -0.2 (-2.3, 1.8) | 14.1 (3.1) | 0.3 (-1.3, 1.9) |
| Total scale Delayed Memory | 96.8 (8.4) | 93.5 (8.3) | -1.4 (-5.4-2.6) | 94.5 (7.0) | -1.6 (-7.3-4.2) | 93.1 (8.8) | -1.3 (-5.7, 3.1) |
| RBANS total scale | 96.4 (10.1) | 92.0 (10.7) | -**5.1 (-9.1, -1.2)*** | 90.4 (6.1) | **-7.8 (-13.5, -2.1)**** | 92.5 (11.9) | **-4.0 (-8.3, 0.4)^** |
| **Additional Tests** |  |  |  |  |  |  |  |
| ***Visual Attention/Reaction Time*** |  |  |  |  |  |  |  |
| Trails Aˠ REVERSE | 23.8 (9.9) | 24.0 (6.8) | -1.8 (-5.5, -1.8) | 27.2 (8.1) | -5.5 (0.5, 10.5) | 22.9 (6.0) | -0.1 (-4.0, 3.8) |
| Trails Bˠ REVERSE | 68.1 (29.1) | 73.7 (28.4) | **-11.5 (-22.5, -0.4)*** | 68.1 (22.1) | -6.4 (-9.3, 22.1) | 75.6 (20.3) | **-13.9 (-26.2, -1.6)** |
| Stroop (I) | 2.0 (10.7) | 0.5 (7.6) | -3.3 (-7.6, 1.1) | -1.1 (7.2) | -4.6 (-10.7, 1.6) | 1.4 (7.6) | -2.7 (-7.5, 2.2) |
| ***Motor speed/Dexterity*** |  |  |  |  |  |  |  |
| Coin rot. Dominant hand | 33.7 (5.3) | 32.2 (6.2) | **-2.8 (-5.3, -0.3)*** | 29.6 (6.8) | -4.0 (-7.5, -0.5) | 33.1 (5.9) | **-2.3 (-5.0, 0.5)^** |
| Coin rot. Non-dominant | 31.3 (5.2) | 28.2 (5.8) | **-3.1 (-5.6, -0.7)*** | 26.0 (5.2) | -4.3 (-7.9, -0.8) | 28.9 (5.9) | **-2.6 (-5.3, 0.2)^** |

**S10 Table. Neuropsychological test scores for Comparison and collision repair workers – Adjusted for both alcohol consumption in the past 48 hours and lifetime alcohol (mean drinks per week).**

^ = p<0.1,* = p<0.05, ** = p<0.01

Adjusted for age, alcohol consumption in the past 48 hours, mean number of alcoholic drinks consumed per week over lifetime, smoking status, DASS A, S and D, test time

(of day) and test day (of week) and premorbid intelligence (NART).

ˠTrails A and B - time to complete each test, therefore higher score represents poorer performance on test – Algebraic sign of coefficient changed accordingly
