# Supplementary material for: Neuropsychological performance in solvent-exposed vehicle collision repair workers in New Zealand
Source: PLoS One. 2017 Dec 13;12(12):e0189108. doi: 10.1371/journal.pone.0189108 (PMC5728539; doi:10.1371/journal.pone.0189108)
Supplement: S11 Table — (DOCX) [file pone.0189108.s011.docx]

|  | Comparison group (n=51) | | Comparison group  previous study (n=160) | | All Collision repair (n=47) | | All Collision repair  Previous study (n=323) | |
| --- | --- | --- | --- | --- | --- | --- | --- | --- |
|  | **n** | **%** | **n** | **%** | **n** | **%** | **n** | **%** |
| **Ethnicity** |  |  |  |  |  |  |  |  |
| Māori | 13 | 25 | 52 | 32 | 3 | 6 | 46 | 14 |
| Pacific | 8 | 16 | 15 | 9 | 2 | 4 | 28 | 9 |
| European New Zealanders and others | 30 | 59 | 93 | 58 | 42 | 89 | 249 | 77 |
| **Smoking Status** |  |  |  |  |  |  |  |  |
| Non-smoker | 18 | 35 | 69 | 35 | 19 | 40 | 151 | 41 |
| Ex-smoker | 16 | 31 | 16 | 31 | 15 | 32 | 95 | 29 |
| Current smoker | 17 | 33 | 17 | 33 | 13 | 28 | 96 | 30 |
| **Lifetime alcohol (frequency)** |  |  |  |  |  |  |  |  |
| Never | 1 | 2 | 8 | 5 | 1 | 2 | 17 | 5 |
| Less than once month | 8 | 16 | 29 | 18 | 4 | 9 | 33 | 10 |
| 1-2 times week | 25 | 49 | 78 | 49 | 25 | 53 | 143 | 44 |
| 3-5 times week | 14 | 27 | 32 | 20 | 16 | 34 | 93 | 29 |
| Daily | 3 | 6 | 13 | 8 | 1 | 2 | 37* | 11 |
| **Education level** |  |  |  |  |  |  |  |  |
| primary school | 0 | 0 | 4 | 3 | 2 | 4 | 8 | 3 |
| secondary school | 36 | 71 | 105 | 66 | 38 | 81 | 223 | 69 |
| trade certification | 9 | 18 | 41 | 26 | 5 | 11 | 80* | 25 |
| Tertiary/University | 6 | 12 | 10 | 6 | 2 | 4 | 12 | 4 |
|  | **Mean** | **Range** | **Mean** | **Range** | **Mean** | **Range** | **Mean** | **Range** |
| **Age** | 39.0 | 19 - 65 | 36.0 | 17 - 66 | 37.8 | 21 - 62 | 36.5 | 17-64 |
| **Lifetime Alcohol (Mean drinks per week)** | 15.8 | 0 - 100 | 15.8 | 0-120 | 14.3 | 0 - 50 | 13.4 | 0 - 140 |
| **Duration of employment (Yrs)** | - | - | - | - | 19.6 | 5.4 - 50.0 | 16.7 | 0.3 - 50 |

**S11 Table. Characteristics of study populations – comparison of demographic characteristics of current study and previous study (**[**1**](#_ENREF_1)**) participants.**

* = p<0.05 (Students t-test)

1. Keer S, Glass B, Prezant B, McLean D, Pearce N, Harding E, et al. Solvent neurotoxicity in vehicle collision repair workers in New Zealand. Neurotoxicology. 2016;57:223-9.
